# Secretome weaponries of *Cochliobolus lunatus* interacting with potato leaf at different temperature regimes reveal a CL[xxxx]LHM - motif

**DOI:** 10.1186/1471-2164-15-213

**Published:** 2014-03-20

**Authors:** Bengyella Louis, Sayanika Devi Waikhom, Pranab Roy, Pardeep Kumar Bhardwaj, Mohendro Wakambam Singh, Sailendra Goyari, Chandradev K Sharma, Narayan Chandra Talukdar

**Affiliations:** 1 Institute of Bioresources and Sustainable Development (IBSD), Takyelpat, Imphal 795001, Manipur, India; 2 Department of Biotechnology, The University of Burdwan, Golapbag More 713104, West Bengal, India; 3 Department of Biochemistry, University of Yaoundé I, Yaoundé-BP812 Yaoundé, Cameroon; 4 Department of Biotechnology, Haldia Institute of Technology, Haldia 721657, West Bengal, India; 5 Regional Centre of the Institute of Bioresources and Sustainable Development (RCIBSD), Gangtok 737102, Sikkim, India; 6 Department of Biotechnology, Guwahati University, Guwahati 781 014, Assam, India

**Keywords:** Thermo-pathogenicity, Candidate effectors, Host-pathogen interaction, Adhesins, Melanized infection hyphae, 2-D electrophoresis

## Abstract

**Background:**

Plant and animal pathogenic fungus *Cochliobolus lunatus* cause great economic damages worldwide every year. *C. lunatus* displays an increased temperature dependent-virulence to a wide range of hosts. Nonetheless, this phenomenon is poorly understood due to lack of insights on the coordinated secretome weaponries produced by *C. lunatus* under heat-stress conditions on putative hosts. To understand the mechanism better, we dissected the secretome of *C. lunatus* interacting with potato (*Solanum tuberosum* L.) leaf at different temperature regimes.

**Results:**

*C. lunatus* produced melanized colonizing hyphae in and on potato leaf, finely modulated the ambient pH as a function of temperature and secreted diverse set of proteins. Using two dimensional gel electrophoresis (2-D) and mass spectrometry (MS) technology, we observed discrete secretomes at 20°C, 28°C and 38°C. A total of 21 differentially expressed peptide spots and 10 unique peptide spots (that did not align on the gels) matched with 28 unique protein models predicted from *C. lunatus* m118 v.2 genome peptides*.* Furthermore, *C. lunatus* secreted peptides via classical and non-classical pathways related to virulence, proteolysis, nucleic acid metabolism, carbohydrate metabolism, heat stress, signal trafficking and some with unidentified catalytic domains.

**Conclusions:**

We have identified a set of 5 soluble candidate effectors of unknown function from *C. lunatus* secretome weaponries against potato crop at different temperature regimes. Our findings demonstrate that *C. lunatus* has a repertoire of signature secretome which mediates thermo-pathogenicity and share a leucine rich “CL[xxxx]LHM”-motif. Considering the rapidly evolving temperature dependent-virulence and host diversity of *C. lunatus*, this data will be useful for designing new protection strategies.

## Background

*Cochliobolus lunatus* (Nelson and Hassis) a member of *Dothideomycetes* predominantly produces four-celled conidia primarily disseminated by air. *C. lunatus* causes several diseases in human [1,2] as well as in food crops such as rice (*Oryza sativa L.*), wheat (*Triticum aestivum*), potato (*Solanum tuberosum* L.), sorghum (*Sorghum bicolor*), cassava (*Manihot esculenta*) and maize (*Zea mays*) [3-8]. Proteomics analysis of virulence variations in *C. lunatus* strains revealed that melanin synthesis-related proteins and heat stress-related proteins (HSP70) are the basic virulence-growth factors during invasion in maize [7,8]. Although only intracellular protein from mycelia was used in these studies [7,8], the data indicated that a large repertoire of functional proteins of *C. lunatus* are unknown.

‘Secretome’ refers to a set of secreted proteins at a given physiologic condition; which plays a key role in cell signaling, intracellular trafficking and migration of invasive weaponries (i.e. candidate effectors) in pathogenic interactions. *C. lunatus* has attracted the interest of many workers on various aspects viz., induce-virulence variation, virulence differentiation and heat-dependent aggressiveness [7-12]. Experimentally, extracellular weaponries secreted by pathogens are crucial for increased virulence and disease development in the context of plant-pathogen interaction sensu stricto. Candidate effector molecules are believed to manipulate host cell structure and function, thereby facilitating infection and suppression of the host immune responses [13,14]. Once candidate effectors are deployed, they act either in the exhaustorial matrix, the extracellular space or within the host cell cytoplasm to promote invasion and pathogenicity [13-15]. In conditions where candidate effectors are recognized by the host disease resistance (*R*) proteins, hallmark resistance occurs via programmed cell death. In this case candidate effectors are considered to have an avirulence activity. Often, fungi discharge their candidate effectors into their surroundings via a non-classical pathway which does not require an N-terminal signal peptide [15]. On the contrary in classical secretory pathway, candidate effectors are directed by the N-terminal peptide signal via the endoplasmic reticulum and Golgi systems to their extracellular locations [13-16].

Frequently, pathogens differentially produce enzymes based on the environmental conditions [16]. Fluctuations of temperature in most cases play a decisive role in the development of disease, since the physiology of either the host or pathogen can change and significantly modulate the interaction dynamics. Interestingly, *C. lunatus* virulence increases with ambient temperature upto 38°C [9-12]. Nevertheless, whether *C. lunatus* discharges secretome weaponries under heat stress conditions on putative host is not known. Thus, examining *C. lunatus* temperature-dependent secretome on a putative host is important and can permit the discovery of candidate effectors that govern its virulence and thermo-pathogenicity. To date, the secretome architecture of *C. lunatus* is not explored and could be of value for designing a suitable control measure in the context of the current rise in global temperature. In this study, microscopic analysis was performed first to decipher the nature of potato leaf invasion in the liquid phase. Subsequently, we used 2-D, MS-technology and *in silico* tools to analyze *C. lunatus* secretome discharged during interaction with potato leaf in the liquid phase. Our work provided first analysis of *C. lunatus* temperature-dependent secretome weaponries deployed during invasion in potato crop.

## Methods

### Plant growth and microorganism culturing conditions

Potato cv. Kufri Jyoti was grown in a plant growth chamber (U-CON250, Danihan Labtech Co., Ltd) at 20°C. The average light intensity was 180 μmolm^-2^ s^-1^ with photoperiod of 16 h light and 8 h darkness. Potato cv. Kufri Jyoti is widely cultivated in India and shows salient resistance features to *Phytophthora infestans* (http://nhb.gov.in/vegetable/potato/pot013.pdf), is moderately susceptible to *C. lunatus *[4] and thermotolerant at 35°C [17]. *C. lunatus* strain btl5 (GenBanK® accession JX907828) was grown on V8 agar medium (Himedia®). The Czapek Dox Broth (CDB) medium composed of 30 g sucrose, 3 g NaNO_3_, 1 g K_2_HPO_4_, 0.5 g MgSO_4_, 0.5 g KCl, 0.01 g FeSO_4_ and 500 mg chloramphenicol in 1 L water was used. The CDB medium was buffered with 100 mM of citric acid-sodium citrate buffer at pH 7.3. Only 10 mm diameter mycelia plug was inoculated in 100 ml CDB medium in a 250 ml conical flask. Five groups of treatments were established as follows. The first control flask contained only *C. lunatus*. Another control flask contained 3 g of disease-free potato leaf fragments devoid of *C. lunatus*. Treatment flasks contained *C. lunatus* and 3 g of disease-free potato leaf fragments. These treatment flasks were incubated independently at 20°C, 28°C and 38°C for 2 weeks under the same photoperiodic conditions that plants were grown and shaken daily at 180 rpm for 10 min.

### Biomass, pH variations and harvest of secreted proteins

After incubation, mycelia and conidia were removed by centrifugation at 13,000 g for 40 min at 4°C. The pH of the supernatant was determined for all the independent replicates using a pH meter (Eutech pH700, ThermoScientific®, Germany). Fresh weight of the interacting complex was measured on a sensitive balance (MicroBalance® C-35, ThermoScientific®, Germany) and 3 g was deducted. The 3 g is assumed to be the equivalent fresh weight of potato leaf added prior to interaction. Next, the complex matter was lyophilized and dry mass was measured in independent replicates. Subsequently, supernatant was chilled at -20°C for 2 h and secreted proteins were isolated by treating the supernatant with 1% sodium deoxycholate (w/v, Sigma®, Saint Louis, USA) and mixed by inversion. The protein complex was precipitated with 15% v/v solution of precipitating agent mixture (100% TCA: 100% acetone, 1:1% v/v) overnight at -20°C. After centrifugation (13,000 g, 30 min, 4°C), protein pellet was washed 5 times with pre-chilled extrapure acetone. Additional cleaning and depigmentation of protein was achieved using clean-up kit (Bio-Rad® laboratories, USA). The precipitates were air-dried for 30 min and dissolved in isoelectric focusing rehydration buffer containing 8 M urea, 2% CHAPS, 50 mM dithiothreitol (DTT), 0.2% (w/v) Bio-Lyte® ampholytes, and bromophenol blue trace (Bio-Rad® laboratories, USA). The protein concentration was determined by the dye-binding method [18]. We used bovine serum albumin for establishing the standard curve and protein aliquots were stored at -80°C till further use.

### Test for leaf invasion in the liquid phase

In order to investigate whether *C. lunatus* established an intimate relationship with potato leaf in CDB medium during interaction, we aseptically removed intact leaf pieces from the reaction flask after 2 weeks of inoculation. Leaves were cleared in glacial acetic acid-ethanol (1:1% v/v) solution at 40°C overnight and rinsed in sterile water with four changes. Here, the chlorazole-black E-KOH staining technique [19] was used for studying the colonization of the abaxial leaf surface. In a randomized block design, we counted necrotic zones every 200 μm^2^ for 10 leaf pieces. Intact leaf pieces were scarce and often difficult to handle for treatment at 38°C. The observation was performed with a microscope coupled with DP7M5.0.0.5 software and an Olympus DP70 camera (Olympus BX61®, USA).

### Data analysis

One-way Anova associated with Tukey’s HSD Post Hoc test were performed to determine the mean significant differences between treatments at *P < 0.05*. Data were computed in SPSS software v.20.

### Two dimensional gel electrophoresis (2-D)

Aliquot of 140 μg of protein sample was used for rehydrating immobilized pH gradient strips (IPG; 7 cm) of pH gradient 4 to 7 (Bio-Rad® laboratories, USA) for 16 h in a passive mode. The pH 4–7 range was predetermined after trials with other focusing range for best resolution. Isoelectric focusing (IEF) was performed at 20°C for a total of 20 KVh using a default rapid ramp protocol on Protean®i12 IEF CELL (Bio-Rad® laboratories, USA). IPG strips were equilibrated twice for 40 min in equilibration buffer I [50 mM Tris–HCl pH 8.8, 6.5 M urea, 30% glycerol (v/v), traces of bromophenol blue and 2% DTT (w/v)] and in equilibration buffer II (50 mM Tris–HCl pH 8.8 and 2.5% iodoacetamide), respectively. The second dimension electrophoresis was performed at 16°C in a Mini-Protean® Tetra Cell (Bio-Rad® laboratories, USA) on a 15% resolving gel. The run was terminated when the dye front reached the lower end of the gel. Gels were calibrated with PrecisionPlusProtein™ WesternC™ Standards (Bio-Rad® laboratories, USA). The gels were stained with Coomassie brilliant blue R250 (CBR) in a solution containing 50% methanol (v/v), 7% glacial acetic acid (v/v) and 0.3% CBR (w/v) overnight at 38°C. Subsequently, gels were destained adequately in a solution containing 30% methanol (v/v) and 7% glacial acetic acid (v/v) until visible spots appeared. Imaging was performed in Molecular Imager Versa DOCMp (Bio-Rad® laboratories, USA).

### Image processing and data analysis

Quality control for gel images and statistical analyses were performed in Progenesis SameSpots v.4.1 suite (TotalLab®, USA). Spots with pixel intensity less than 120, spots in damaged areas and at the edge of the gel were excluded prior to nonlinear dynamics alignment. Spot volume (pixel-by-pixel intensity) were normalized as parts per million (ppm) of the total spot volume to determine the fold expression. Importantly, results were validated by performing pixel-to-pixel correlation analysis with an Anova *P*-value ≤0.05 at a fold expression cut-off value (*F*) ≥1.0. Here, differentially expressed spots and unique spots that did not align, judged not to be false positive based on eight gel runs were manually excised for downstream analysis.

### Protein digestion and mass spectrometry

Protein digestion was performed as previously described [20]. Briefly, 0.45 μl of digested protein solution was mixed in 0.45 μl of α-cyano-4-hydroxycinnamic acid solution on matrix-assisted laser desorption/ionization time-of-flight/time-of-flight mass spectrometry (MALDI-TOF/TOF MS) 4800 proteomics analyzer targeted plate. Peak lists were processed and exported through 4000 Series Explorer Software (Applied Biosystem, MA, USA) at default settings. Homology search was performed using MASCOT v.2.3 (MatrixScience, London, UK) through Proteome Discoverer v.1.3.0.339 (ThermoScientific, Germany) against filtered predicted protein models (originated from expression sequence tag) from *C. lunatus* 20120521 m118 v2.0 genome peptides available at http://genome.jgi.doe.gov. The search parameters were: Enzyme, trypsin; Fixed modifications, carbamidomethyl (C); Variable modification, oxidation (M); Peptide mass tolerance, 40–100 ppm; Maximum missed cleavages, 2. The accepted MOWSE score threshold was inferred at *P < 0.05*. A few peptide peak lists that failed to match *C. lunatus* m118 v2.0 model genome peptides were queried against all updated entries from the NCBInr and Fungi MSDB sequence databases via in-house MASCOT server (v.2.3 MatrixScience, London, UK) using identical search parameters. In case of homologous proteins having similar MOWSE scores, we gave preference to proteins with best matched theoretical and experimental p*I*. False-discovery rate (FDR) [21] for the peptide search match was calculated using a decoy database (http://www.matrixscience.com/help/decoy_help.html). Here, we set FDR of 1% as a cut-off to export results from the analysis. Among the 60 spots excised from the gels that were analysed, only 39 were validated at FDR ≤1% and reported. Each step in the identification process was verified manually.

### *In silico* characterization of secretome and *de novo* motif searches

Signal peptide was predicted in SignalP 4.1 server [22]. A cut-off discriminatory score (D) was used to discern signal peptide with or without transmembrane (TM) network as follows. D = 0.45 for signal peptide without TM network and D = 0.50 for signal peptide with TM network. Subcellular localization of candidate effectors target was predicted at default settings in TargetP v.1 server [23]. Theoretical p*I* was predicted as earlier described [24] and theoretical molecular weight and protein net charge was predicted at http://www.encorbio.com/protocols/Prot-MW.htm. Glycosylphosphatidylinositol (GPI) anchored was predicted in big-pi web server [25]. Adhesin was predicted in Faapred [26] and FungalRV [27] web servers. Motif search was performed in MEME v.4.9 suite [28]. Here, we used sequences from *C. lunatus* model proteins only (elaborated in Additional file 1: Fasta) with significant hits (E-value <10^-5^). The search was set to account for a maximum of 3 different motifs with 4 to 10 amino acids width with shuffling option. The motifs dispersion was set at zero or one per sequence. Identified motifs at *P*-value <10^-5^ were retained and queried against GenBank® 23 fungal genomes peptides to determine the best likelihood matches using MAST v.4.9 suite [29]. The fidelity of the best hit motif was confirmed by scanning for the occurrences in 23 fungal genomes peptides using FIMO v.4.9 [30].

### RNA isolation and gene expression analysis of candidate effectors

After a quick separation of mycelia mat from the leaf fragments at the same time points, total RNA was isolated from mycelium using TRIzol® reagent (Invitrogen, USA) following the manufacturer’s protocol. Total RNA (1 μg) was treated with 1U RNAse-free DNase I (Amplification grade; Invitrogen, USA) and used in subsequent steps. The concentration of total RNA was determined by spectrophotometer (BioSpec-nano, Shimadzu®, Japan) and the integrity was assessed by performing denaturing formaldehyde-agarose gel electrophoresis. Gene expression analysis was performed by semi-quantitative reverse transcriptase-polymerase chain reaction (RT-PCR). The first-strand complementary DNA (cDNA) was synthesized using SuperScript® VILA™ cDNA synthesis kit (Invitrogen, USA) following the manufacturer’s protocol. PCR was carried out using gene specific primers and expression was evaluated at the exponential phase of amplification. In brief, PCR was performed using 1 μl of cDNA template (diluted 10 times), 0.2 mM of dNTPs, 0.2 μM each of forward and reverse primers, 1 U of GoTaq® DNA Polymerase (Promega, USA) and 1X PCR buffer (Promega, USA) in a final volume of 25 μl. The thermal cycling conditions were 94°C for 4 min, 25–30 cycles of 94°C for 30 s, 55-56°C for 30 s, 72°C for 30 s and a final extension at 72°C for 7 min. Expression of *glyceraldehyde 3-phosphate dehydrogenase* (*GAPDH*) gene was used as internal control to equalize cDNA quantities in all the reactions. All the primer sequences and annealing temperatures are elaborated (Additional file 2: Table S1).

## Results

### *C. lunatus* produced large biomass and modified the ambient pH

**Figure 1:**
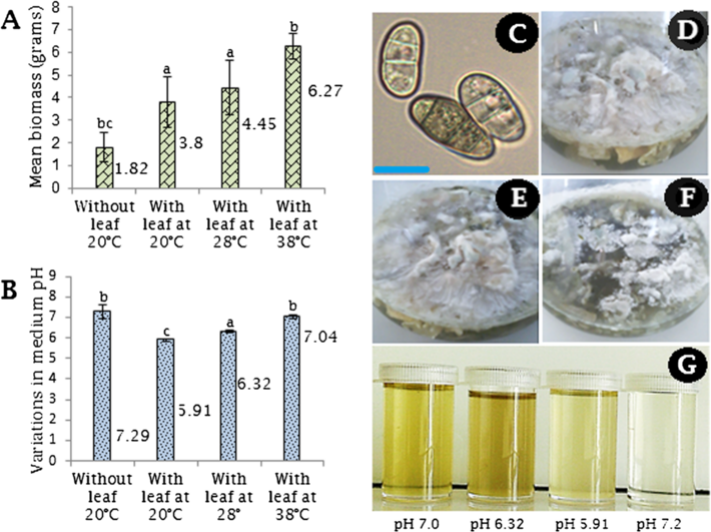
**Culture conditions for *Cochliobolus lunatus *strain btl5 (GenBank**^**® **^**accession JX907828) following two weeks of inoculation in Czapek Dox Broth medium. (A)** Variations in biomass. **(B)** Variations in medium pH. Bars represent standard errors of the mean, and ^a,b,c,bc^denotes mean treatments that are significantly different according to Tukey’s HSD post-hoc test at *P < 0.05*. **(C)** Micrograph of conidia harvested at 20°C with leaf and scale bar is 20 μm. **(D)** Culture flask with potato leaf at 20°C. **(E)** Culture flask with potato leaf at 38°C. **(F)** Culture flask without potato leaf at 20°C. **(G)** From left to right, supernatant of cultures medium obtain at 38°C with leaf, 28°C with leaf, 20°C with leaf and 20°C without leaf.

*C. lunatus* produced floccose mycelia mat in CDB medium. Importantly, interaction of *C. lunatus* with potato leaf at 38°C produced the maximum significant biomass (6.27 g*, P < 0.003, F = 18.08*). Lowest biomass (1.82 g, *P < 0.003, F = 18.08*) was scored in the absence of potato leaf at 20°C (Figure 1A). Curiously, significant changes in pH i.e. from 7.30 to 5.91 (*P = 0.00, F = 102.19*) was observed between *C. lunatus-*leaf interaction at 20°C. In this study we selected 20°C because potato is a winter crop. Other temperatures such as 25°C, 28°C, 35°C and 38°C were tested in standardizing the experiment. When we compared the secretome maps of 25°C, 28°C, 35°C and 38°C with that of 20°C treatment in the presence of potato leaf, only 38°C produced a unique pattern of peptide spots. Therefore, we focused on 38°C as optimal heat-stress condition for downstream secretome analysis. The heat stress was aimed at providing an intuitive insight on the thermo-pathogenic nature of *C. lunatus vis-à-vis* potato host. Interestingly, in flask with potato leaf at 38°C, only a slight change in medium pH i.e. from 7.30 to 7.04 was scored against a significant biomass produced (6.27 g, *P < 0.003, F = 18.08*: Figure 1A, B). The result shows that *C. lunatus* modified the ambient pH during interaction with potato leaf as a function of temperature. That is, *C. lunatus* proliferated by changing the neutral medium (pH 7.30) to: 1) acidic medium (pH 5.91) at 20°C with potato leaf and 2) maintained a neutral medium (pH 7.04) at 38°C with potato leaf. This ability to modulate the pH of the interaction medium enables *C. lunatus* to secrete different set of candidate effectors. This was phenotypically manifested in the variable colors of conidia (Figure 1C) and variable colors of the culture medium (Figure 1D-G). No change in pH was observed in the control flask containing potato leaf without *C. lunatus* (data not shown), and we also failed to harvest proteins from the liquid medium.

### *C. lunatus* invaded potato leaf in the liquid phase

**Figure 2:**
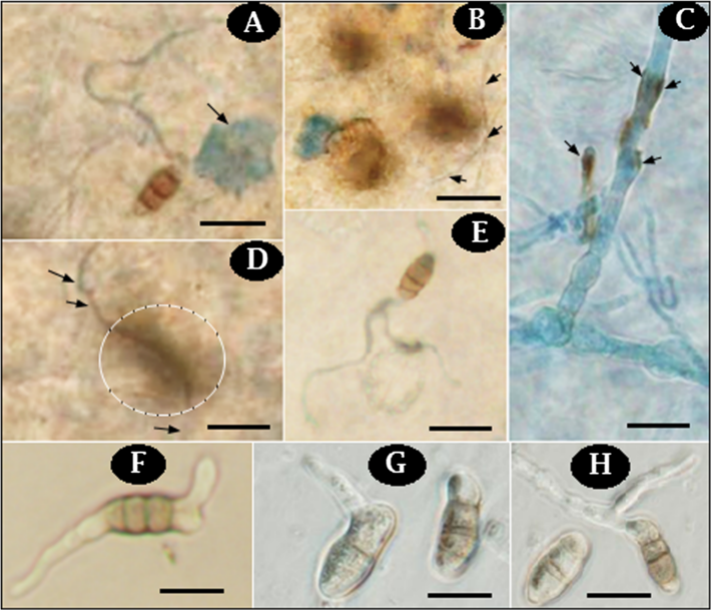
***Cochliobolus lunatus *invaded potato leaf in CDB medium. (A)** Arrow indicates dead epidermal cells (DEC) at 20°C, 400X and scale is 20 μm. **(B)** Arrows indicate subcutaneous penetration of epidermal cells at 38°C, 400X and scale is 20 μm. **(C)** Arrows indicate pigmented hypha on the leaf surface at 38°C, 1000X and scale is 20 μm. **(D)** Arrows indicate an infectious hyphae penetrating subcutaneously and causing necrotic zones (encircled) at 28°C, 1000X and scale is 20 μm. **(E)** Colonizing conidia spreading on the abaxial leaf surface at 28°C, 400X and scale is 20 μm. **(F)** Bipolar conidium germination at 20°C without potato leaf, 400X and scale is 20 μm. **(G)** Germinating conidia at 28°C in CDB with potato leaf, 1000X and scale is 20 μm. **(H)** Germinating conidia at 38°C in CDB with potato leaf, 1000X and scale is 20 μm.

**Figure 3:**
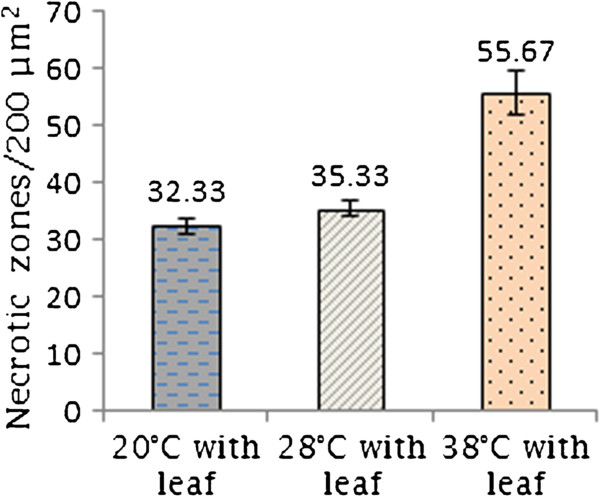
**Mean value of necrotic zones counted on a 200 μm**^**2 **^**per leaf surface for ten observations at *P < 0.05 *** **and bars represent standard errors of the mean** ***.***

*C. lunatus* penetrated the leaf tissues during interaction. No appressoria-like structure was observed. Remarkably, the infectious hyphae penetrated the leaf tissues subcutaneously, forming necrotic epidermal zones (Figure 2), dead epidermal cells (Figure 2A) and pigmented hyphae on the leaf surface (Figure 2B,C). Worth mentioning, the infectious melanized hyphae of *C. lunatus* at 20°C often penetrated via the epidermal anticlinal cell wall killing the surrounding cells as manifested by brown-to-black tissue (Figure 2D). Germinating conidia on the leaf-CDB interface (Figure 2E) and germinating conidia in CDB medium (Figure 2F-H) were often observed. As shown (Figure 2), two weeks after inoculation, colonization was an active ongoing process and *C. lunatus* intimately interacted with the potato leaf. A mean of 32.33 necrotic zones per 200 μm^2^ of leaf tissue (*P < 0.05; F = 8.57*) was scored at 20°C. At the other extreme temperature, 57.67 necrotic zones per 200 μm^2^ of leaf tissue (*P < 0.05; F = 3.10*) was scored at 38°C (Figure 3).

### *C. lunatus* produced diverse repertoire of secretomes

**Figure 4:**
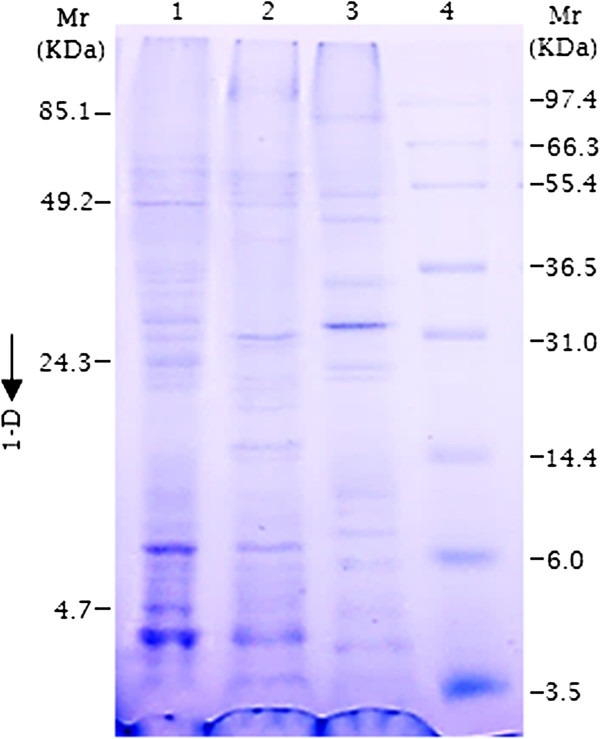
**Secretome profile obtained for the interaction of *Cochliobolus lunatus *with potato leaf in CDB medium.** Proteins were separated on a 1-D gel (4:15)% w/v and stained with CBR. Lane 1, 2 and 3- (from left) are secreted proteins obtained at 20°C, 28°C and 38°C, respectively. Lane 4 - represents standard molecular weight marker.

In the course of standardizing this experiment, we found that addition of 1% sodium deoxycholate (w/v) to the filtered CDB interaction medium without chilling at -20°C resulted in: 1) difficulties in eliminating pigments, 2) low number of distinct peptide spots, and 3) vertical striking on the gel. Hence, the interaction medium was chilled at -20°C for 2 h before treatment with 1% sodium deoxycholate (w/v). Importantly, when proteins were fully dried by lyophilization, we faced difficulties in re-solubilizing the samples. Therefore, in this protocol we do not recommend lyophilization of the protein sample prior to re-suspension in 2-D rehydration buffer (Bio-Rad® laboratories, USA). In order to check the quality of our extraction procedure we performed a one dimensional gel electrophoresis (1-D). The 1-D profile of *C. lunatus* total secretome interacting at 20°C with potato leaf, 28°C with potato leaf and 38°C with potato leaf indicated a differential expression pattern (Figure 4). This result suggested that *C. lunatus* adapts to different temperature regimes during interaction with potato crop by differentially expressing diverse set of secretome (Figure 4).

**Figure 5:**
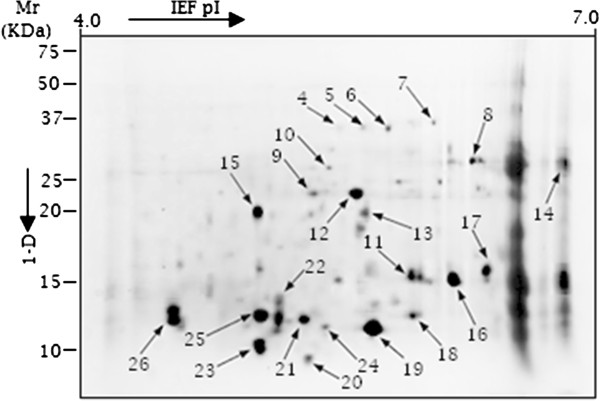
**A 2-D secretome map of *Cochliobolus lunatus *interacting with potato leaf in CDB medium cultured at 20°C*.*** Identified spots are indicated with arrows. The immobilized pH gradient scale and standard molecular weight scale are shown.

**Figure 6:**
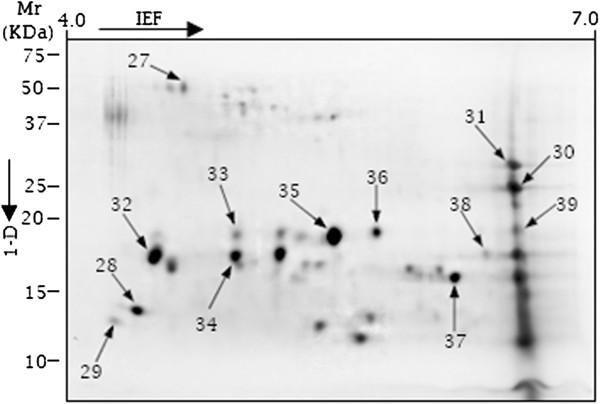
**A 2-D secretome map of *Cochliobolus lunatus *interacting with potato leaf in CDB medium cultured at 38°C*.*** Identified spots are indicated with arrows. The immobilized pH gradient scale and standard molecular weight scale are shown.

**Figure 7:**
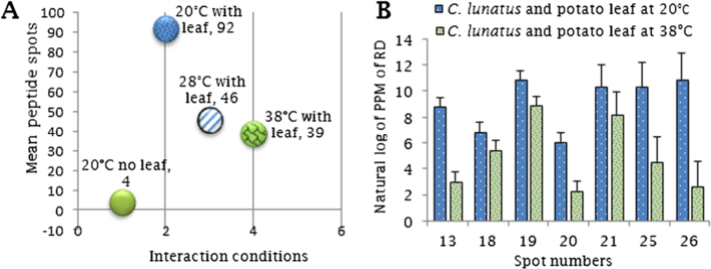
**Graphical representations of secretome changes. (A)** An overview of peptide spots detected on secretome maps at different temperature regimes. **(B)** The most significant differentially up-regulated peptide spots at 20°C and 38°C in the presence of potato leaf expressed in part per million (ppm) of relative density (RD) based on correlation analysis at Anova *P* ≤ 0.05 and bars represent standard errors of the mean values.

Following normalization of 2-D gels in Progenesis SameSpotv.4.1, the following reproducible peptide spot counts were present in at least 3 replicates per treatment: 1) 4 peptides spots without potato leaf (Additional file 3: Figure S1), 2) 92 peptide spots at 20°C in the presence of potato leaf (Figure 5), 3) 46 peptide spots at 28°C in the presence of potato leaf (Additional file 4: Figure S2) and 4) 39 peptide spots at 38°C in the presence of potato leaf (Figures 6 and 7A). The plot showing positional normalized spot volumes on the gels are depicted (Additional file 5: Figure S3). As shown (Figure 7A), *C. lunatus* differentially expressed less peptide spots at 38°C. A comparative analysis of the secretome maps for the treatment with potato leaf at 20°C and 38°C revealed that peptide spots# 11, 12, 28, 29, 30, 31, 32 and 39 were unique (Figures 5 and 6). Thus, these peptide spots did not align in all gels (8 gels, 4 biological replications for each treatment, data not shown). Peptide spots# 28, 29, 30, 31, 32 and 39 were only observed at 38°C (Figure 6). Overall, we observed that abundant peptide spots were of low molecular mass (Mr) ranging from 9.33-46 KDa; and having an experimental p*I* ranging from 4.26 to 6.62 on a 4–7 p*I* scale. Key features like homology, putative function etc. for the identified peptide spots are elaborated (Table 1: Additional file 6: Table S2A, B).

**Table 1:** **Identification, expression pattern, and functional characterization of differentially secreted proteins in *Cochliobolus lunatus *** **at different temperature regimes**

**Spot #**	^**a**^**Diff exp.**	**Protein hits**	^**b**^**Th. p *I */**^**c**^**Exp. p** ***I***	^**d**^**Th. Mr/**^**e**^**Ex. Mr. / **^**f**^**Net Char.**	^**g**^**M. Sc.**	^**h**^**Seq. Cover.**	**Putative function**	**Interaction conditions**
1	Up-regulated	123093|e_gw1.16.937.1	5.7/6.5	39.6/18.2/-4	182	49	Protein kinase activity	No potato leaf at 20°C
2	Up-regulated	16455|fgenesh1_pg.2_#_314	9.7/6.5	64.0/15.6/14	179	56	Modulate transcription	
3	Up-regulated	118437|e_gw1.13.184.1	10.7/6.6	18.8/11.87/18	398	45	Unknown	
4	Up-regulated	125544|e_gw1.19.437.1	7.2/5.5	58.7/42.4/4	365	46	Oxido-reductase activity	
5	Up-regulated	24606|fgenesh1_pg.19_#_39	10.2/5.6	110.2/45.4/46	169	43	Protein kinase activity	
6	Up-regulated	19257|fgenesh1_pg.7_#_229	6.2/5.8	195.0/46.3/-1.5	299	33	Unknown	
7	Up-regulated	115068|e_gw1.10.331.1	4.2/6.0	261.8/45.2/-157	294	23	Heat shock protein 70kD	
8	Up-regulated	129342|estExt_Genewise1.C_1_t20028	6.7/6.2	195.7/36.4/9	179	38	RNA binding binding protein	
9	Up-regulated	115068|e_gw1.10.331.1	4.5/5.3	261.8/35.1/-157	279	31	HSP70	
10	Up-regulated	129342|estExt_Genewise1.C_1_t20028	6.7/5.4	195.7/32.3/9	110	43	RNA processing	
11	Up-regulated	19962|fgenesh1_pg.8_#_384	6.3/5.9	181.8/31.5/1	189	29	Protein kinase activity	
12	Up-regulated	116559|e_gw1.11.1289.1	6.7/5.6	114.9/24.3/7.5	275	25	Subtilisin, proteolysis	
13	Up-regulated	21721|fgenesh1_pg.12_#_162	9/5.6	146.7/21.4/24	161	26	Pleckstrin-like, intracellular signalling	
14	Up-regulated	22022|fgenesh1_pg.12_#_463	10.4/6.8	27.5/16.0/18.5	382	32	Ribonuclease activity	
15	Up-regulated	116337|e_gw1.11.1021.1	10.1/6.1	183.2/22.8/59	497	40	Gelsolin-like intratrafficking protein	
16	Up-regulated	51954|fgenesh1_pm.10_#_434	10.1/6.1	63.9/16.0/20.5	340	40	DNA binding protein	
17	Up-regulated	140405|estExt_Genewise1.C_210317	6.2/6.3	91.9/12.8/91.9	245	28	Arrestin-like signaling protein	
18	Up-regulated	137106|estExt_Genewise1.C_13_t10496	4.9/5.9	126.1/14.53/-31.5	198	32	Tetratricopeptide intracellular trafficking	
19	Up-regulated	46252|estExt_Genemark1.C_210087	10.0/5.7	52.3/12.3/20.5	287	22	Unknown	
21	Up-regulated	64448|estExt_fgenesh1_pg.C_160063	9.9/5.3	112.8/13.1/53	111	29	Unknown	
22	Up-regulated	59412|estExt_fgenesh1_pm.C_130013	7.2/5.3	109.9/13.4/12.5	100	34	WD40 repeat, signal transduction	
23	Up-regulated	29439|fgenesh1_kg.13_#_117_#_Contig_2	7.1/5.0	28.6/10.7/3	276	39	Short-chain dehydrogenase/reductase	
24	Up-regulated	58690|estExt_fgenesh1_pm.C_9_t10419	5.2/5.4	88.5/11.3/-14	369	21	Proteolysis	
25	Up-regulated	125654|e_gw1.19.739.1	5.7/5.0	145.5/13.4/-9.5	476	24	Signal transduction response regulator	
27	Up-regulated	132047|estExt_Genewise1.C_4_t40098	9.4/4.6	44.7/71.0/6.5	282	49	Unknown	Potato leaf at 20°C
28	Induced	123093|e_gw1.16.937.1	5.7/4.4	39.6/18.3/-4	165	21	Protein kinase activity	
29	Induced	16455|fgenesh1_pg.2_#_314	9.7/4.2	64.0/14.1/14	186	38	DNA binding transcription factor	
30	Induced	114571|e_gw1.10.1404.1	6.6/6.5	49.0/29.0/4	188	22	Carbohydrate metabolism	
31	Induced	141118|estExt_Genewise1Plus.C_1_t20192	4.7/6.5	89.9/35.3/-26.5	359	28	Nucleic acid metabolism	
32	Induced	44339|estExt_Genemark1.C_10_t10268	5.9/4.5	106.7/20.63/-6.5	162	29	Carbohydrate metabolism	
38	Induced	46026|estExt_Genemark1.C_180273	11.2/6.2	21.8/18.6/1.5	195	31	Cytochrome C oxidase	Potato leaf at 38°C

^**#**^Spot number corresponds to the spots in Additional file 3: Figure S1, Figures 5 and 6. ^**a**^Differential protein expression, ^b^Theoretical isoelectric point (p*I*), ^c^Experimental p*I*, ^d^Theoretical molecular mass based on whole protein sequence, ^e^Experimental molecular mass of protein subunits in KDa, ^f^Predicted protein net charge based on whole protein sequence, ^g^Protein score reported by MASCOT MS Ion search, and ^h^Amino acid sequence coverage. This group of proteins was used for motif searching by *in silico* tools. All the proteins were identified by homology search at http://genome.jgi.doe.gov/Coclu2/Coclu2.home.html.

Next, we performed correlation analysis with peptide spots that aligned to determine the changes in their expression using Progenesis SameSpot v.4.1. This analysis was important since strong correlation indicated how these peptides are co-regulated and therefore, their involvement in virulence during invasion. Significantly expressed peptide spot# 13 (P = 2.e^-0.6^, 2.3 folds, 21.48 KDa) was identified as pleckstrin-like related protein (Table 1). Peptide spot# 18 (P = 0.009, 2.3 folds, 14.53 KDa) was identified as tetratricopetide (TPR)-like related protein (Table 1). Peptide spot# 25 (P = 2.39.e^-0.04^, 6 folds, 13.48 KDa) often plays a large role in signal transduction response regulation (Table 1). Nonetheless, peptides spot# 19 (P = 0.013, 3.3 folds, 12.33 KDa), spot# 20 (P = 0.025, 2.3 folds, 9.67 KDa) and spot# 21 (P = 0.033, 3.8 folds, 13.17 KDa) failed to have a known catalytic domain (Table 1: Additional file 6: Table S2A, B). The profile for differentially expressed peptide spots# 13, 18, 19, 20, 21, 25 and 26 and their accessions in *C. lunatus* m118 v.2 genome peptides is depicted (Figure 7B: Table 1: Additional file 6: Table S2A).

**Figure 8:**
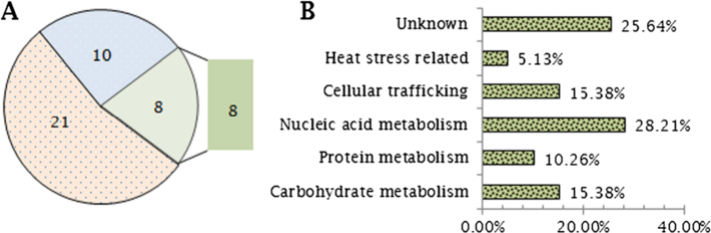
**Graphic summations of identified peptide spots. (A)** 8 peptide spots, matching in gels did not match with *C. lunatus* filtered model proteins. 10 mismatched peptide spots and 21 matched peptides spots in gels matched *C. lunatus* filtered model proteins. **(B)** The relative categorization of secreted proteins identified show the majority is involved in nucleic acid metabolism and some with unknown functions.

In the process of search performed against unique protein models predicted from *C. lunatus* m118 v.2 model genome peptides, 31 out of the 39 identified peptide spots produced 21 unique significant hits (*E-value* <10^-5^) and the profile for the 10 best hits is shown (Additional file 7: Figure S4). 5 out of the 31 peptide spots were without known functions. 8 out of 39 peptide spots failed to match with unique protein models from *C. lunatus* m118 v.2 model genome peptides. Of the 8 peptide spots, we performed combined searches in fungi MSDB and NCBInr data bases. Once more, 5 out of 8 peptide spots were without known putative functions (Additional file 6: Table S2B). Based on the putative functions of the identified peptides, 10 were unknown (8 up-regulated, 1 induced, 1 unique spot), 6 carbohydrate active-enzymes (3 up-regulated, 3 unique spots), 2 heat shock related proteins (1 up-regulated, 1 unique spot), 6 cellular traffickers (6 up-regulated), 4 protease/proteolysis related-proteins (3 up-regulated, 1 unique spot) and 11 nucleic acid metabolism related (5 up-regulated, 6 unique spots) and their relative abundance is represented (Figure 8A,B). Importantly, the peptides spot# 12, 24 and 26 that were significantly expressed had putative proteolytic activity. HSP70-like related proteins were peptide spot# 7 (P = 0.568; 1.2 folds; 45.23 KDa) and peptide spot# 9 (P = 0.445; 1.9 folds; 35.10 KDa).

### *C. lunatus* used both classical and non-classical secretory pathways

Using SignalP4.1server [22], we found peptide spot# 38 (Cytochrome C oxidase related protein) was secreted through conventional pathway having an N-terminal signal peptide and forming TM-networks only (Additional file 6: Table S2A: Additional file 8: Figure S5). To address the potential destination of the proteins, Target P1.1 [23] algorithm was used. We found that 12 peptide spots could target the mitochondrion, 16 peptide spots could target any subcellular organelle and 11 peptide spots had no predictable targets (Additional file 6: Table S2A, B). Throughout this study, no protein was predicted to have GPI-anchor motif (data not shown). This was a confirmation that the proteins observed in the secretome were not an outcome of fortuitous discharge from fungal cell surface or leaf tissue. Only 10 out of 31 proteins that matched unique protein models predicted from *C. lunatus* m118 v.2 genome peptides were found to have adhesin motifs (Additional file 6: Table S2A, B). Overall, only 10 out of 31 unique protein models from *C. lunatus* m118 v.2 model genome peptides identified in this study had a net negative charge (Table 1). This data revealed that proteins secreted during colonization of potato by *C. lunatus* are mostly positively charged.

### *C. lunatus* secretomes on potato leaf contained a CLxxxxLHM-motif

**Figure 9:**
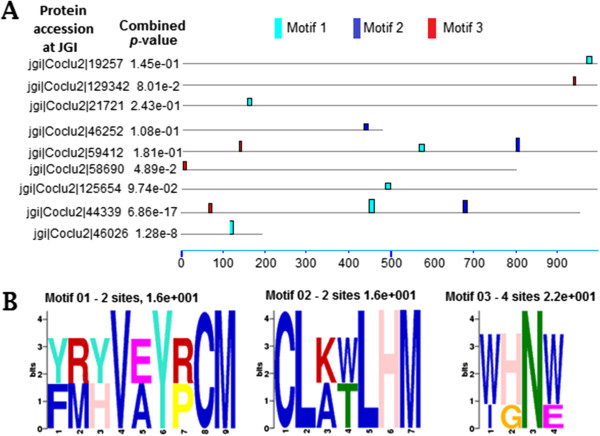
***De novo *motif searches in *Cochliobolus lunatus *secretome reveal conserved hydrophobic amino acid residues reported in MEME 4.9 **[28]** algorithm. (A)** A motif block diagram showing motifs distribution secreted effector indicated by bars. **(B)** Sequence logos of 3 motifs of a maximum of 14 amino acids length with highest non-positional constraint likelihoods scattered over the secreted effector sites. The height of the motif “block” is proportional to -log (*P*-value), truncated at the height for a motif with a *P-*value of 1e^-10^. Blue indicates the most hydrophobic residue; green indicates the most polar, non-charged, non-aliphatic residue; magenta indicates the most acidic; and red indicates the most positively charged residue.

**Figure 10:**
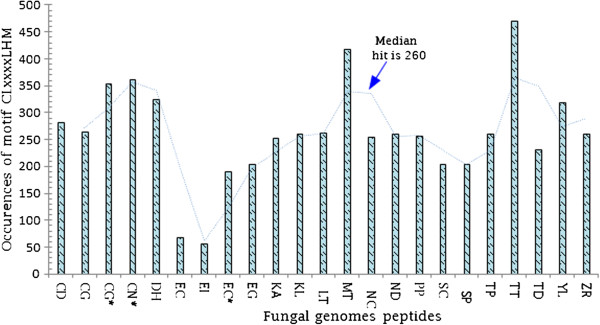
**Occurrences hits for motif CLxxxxLHM in 23 fungal genomes peptides present in the GenBank**^**® **^**as evaluated in FIMO v.4.9 **[30]**produced a median hit score of 260 (dotted line) at *p < 0.001*.**

Inspired by the lack of putative catalytic domains in some candidate effectors, we searched for conserved motifs in MEME v.4.9 [28]. We found that motifs [FY][MR][HY]V[AE]Y[PR]CM, CL[AK][TW]LHM and [WI][HG]N[WE] were distributed on peptides sequences with non-overlapping sites (*P*-value < 10^-5^). Conserved amino acid residues in motif 01 were V, Y, C and M and organized in an xxxxxxVxxYxxCM pattern dispersed over the N-terminal (Figure 9A). ‘X’ represents any variable amino acid residue in the pattern. Motif 02 contained C, L, L, H and M conserved amino acid residues organized in a CLxxxxLHM pattern and distributed within the range of 450 to 830 amino acids residues after the N-terminal cleavage site. Motif 03 contained a major conserve N (or asparagine) residue, W, H and W with four possible sites. This motif could be organized as xWxHNxW. Amino acids organization is elaborated in the motif logos (Figure 9B). We tested the strength of motifs against GenBank® 23 fungal genomes peptides, essentially, cross kingdom pathogens such as *Candida dubliniensis CD36* uid38659 (CD), *Candida glabrata* (CG), *Cryptococcus gattii* WM276 (CG*), *Cryptococcus neoformans var* JEC21 uid10698 (CN), *Debaryomyces hansenii* CBS767 uid12410 (DH), *Encephalitozoon cuniculi* uid155 (EC), *Encephalitozoon intestinalis* ATCC 50506 uid51607 (EI), *Eremothecium cymbalariae* DBVPG 7215 (EC*), *Eremothecium gossypii* uid10623(EG), *Kazachstania africana* CBS 2517 (KA), *Kluyveromyces lactis* NRRL Y-1140 uid12377(KL), *Lachancea thermotolerans* CBS 6340 uid39575(LT), *Myceliophthora thermophila* ATCC 42464 (MT), *Naumovozyma castellii* CBS 4309 (NC), *Naumovozyma dairenensis* CBS 421(ND), *Pichia pastoris* GS115 uid39439 (PP), *Saccharomyces cerevisiae* uid128 (SC), *Schizosaccharomyces pombe* uid127 (SP), *Tetrapisispora phaffii* CBS 4417 (TP), *Thielavia terrestris* NRRL 8126 (TT)*, Torulaspora delbrueckii* CBS 1146 (TD), *Yarrowia lipolytica* CLIB122 uid12414 (YL) and *Zygosaccharomyces rouxii* CBS 732 uid39573 (ZR) to determine the best motif fidelity using MAST v.4.9 [29] and occurrence rate using FIMO v.4.9 [30]. Motif 02 (CLxxxxLHM) produced the best fidelity match and occurrence hits (Additional file 9: Table S3). The median occurrence score of motif 02 was 260 per fungal genome peptides. Furthermore, motif 02 maximum and minimum hits of 470 and 56 was scored in *Thielavia terrestris* NRRL8126 and *Encephalitozoon intestinalis ATCC* 50506uid5160 genome peptides, respectively (Figure 10).

### Expression profiling of genes encoding secreted candidate effector proteins

**Figure 11:**
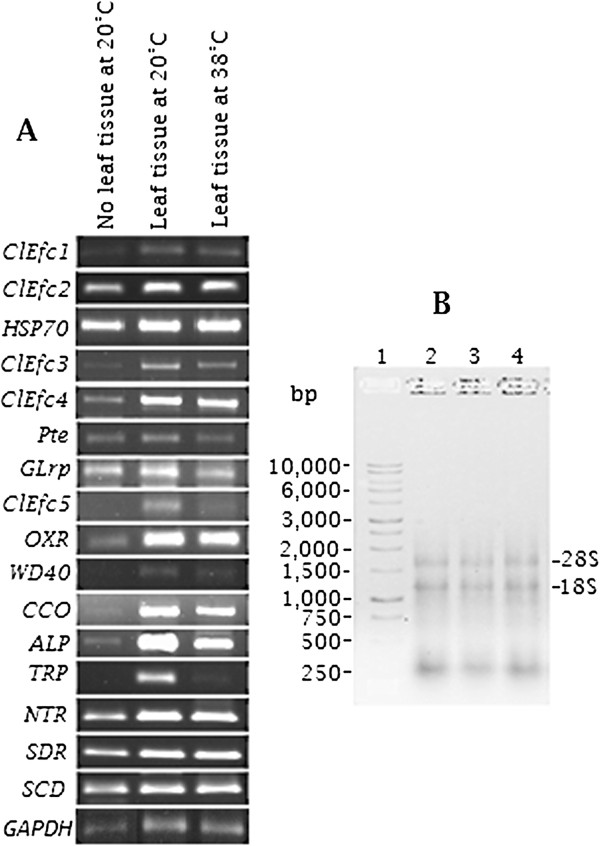
**Semi-quantitative reverse transcriptase-polymerase chain reaction (RT-PCR) analysis. (A)** Gene expression analysis for *Cochliobolus lunatus* effector candidates (*ClEfc*) and virulence-related candidate effectors. **(B)***C. lunatus* total RNA following DNase 1 treatment separated on a 2% denaturing formaldehydeagarose gel electrophoresis. Lane 1 (from left) represents 1 kb DNA ladder (Promega^®^, USA). Lane 2, 3 and 4 are total RNA extracted from mycelium grown at 20°C without potato leaf, 20°C with potato leaf and 38°C with potato leaf at the same time point, respectively. Expression of *C. lunatus GAPDH* was used as internal control.

The identification of differentially expressed peptide spots with unknown catalytic domains and other virulence-related peptides prompted us to analyze their gene expression using semi-quantitative RT-PCR. Consistent and reproducible differential expressions were obtained for 16 candidates genes (Figure 11A). The 5 unique putative *C. lunatus* effector candidates (*ClEfc1, ClEfc2, ClEfc3, ClEfc4 and ClEfc5*) with unknown catalytic domains were expressed at both 20°C and 38°C in the presence of potato leaf. Virulence related genes namely *heat shock protein 70* (*HSP70*), *scytalone dehydratase* (*SDR*), *1,3,8-naphthalenetriol reductase* (*NTR*), *tetratricopeptide* (*TRP*), *oxidoreductase* (*OXR*), *subtilisin protease* (*Pte*), *WD40 repeat signalling peptides* (*WD40*), *arrestin-like protein* (*ALP*), *short chain dehydrogenase* (*SCD*), *cytochrome C oxidase* (*CCO*) and *gasolin-like related protein* (*GLrP*) were differentially up-regulated during potato leaf colonization (Figure 11A). Total RNA profile is shown (Figure 11B).

## Discussion

A great challenge in secretomics is how to trigger a pathogen to secrete proteins as starting material for downstream analysis. Studies based on genome scanning for secretome through *in silico* approach is less cumbersome and generates large data [15,25-28]. Nevertheless, the likelihood that predicted proteins expressed are during host colonization is very low. Moreover, the physiological conditions for secretion cannot be predicted *in silico*.

During matrix-assisted laser desorption/ionization time-of-flight/time-of-flight mass spectrometry (MALDI-TOF/TOF MS) we found that several peptide spots showed incongruities between their experimental and predicted molecular mass (Mr) and *pI*. These inconsistencies in experimental and theoretical values of *pI* and molecular masses have been observed in several proteomics studies [31-34]. While predicted values are based on whole protein sequence, experimental values are a function of protein subunits only detected on gels. Ecological niche can cause dramatic changes in experimental *pI* of proteins from acidic *pI* to basic *pI *[32]. Interestingly, it is reported that *in vivo* or *in vitro* protein degradation can result in experimental Mr significantly lower than predicted Mr [34]. This might hold true for this study given *C. lunatus* secreted proteolytic proteins (Table 1). Furthermore, post-translational modifications such as ubiquitination, sumoylation, glycosylation, alternative splicing and endoproteolytic cleavage can cause discrepancies in predicted and expected *pI* and Mr values [33,35].

We found that *C. lunatus* modified the ambient pH. Some fungi have developed complicated regulatory mechanism to sense and respond to ambient pH dependent signals during prolonged interaction with the environment [36]. In addition, a few fungi secrete different set of proteins at different ambient pH [37]. For a successful invasion of a host, pathogens require coordinated secretion of chemical weaponries at the onset of interaction. For instance, an important hemibiotroph such as *Botrytis cinerea* differentially secretes enzymes in various plant tissues during invasion [16]. Cooperation of various extracellular proteins, though a few may be ‘real’ virulence factors [37] is decisive for the successful suppression of the host defense response. The changes in biomass and pH observed in this study at different temperature regimes demonstrated that *C. lunatus* successfully thrived on potato leaf by finely adjusting its microenvironment (Figure 1A,B).

The secretome studies of *B. cinerea* revealed a dark colored culture medium at pH 4 and pH 6 [37]. This dark color was due to secondary metabolites produced during the fungus growth [37]. In the present secretome analysis, the intense yellowish- and milky-colored culture medium (Figure 1D-G) and melanized colonizing hyphae (Figure 2) indicated that *C. lunatus* secretome comprises of colored secondary metabolites along with their candidate effectors. Previous study revealed that *C. lunatus* strains which produced high level of melanin are more virulent than those producing low level melanin [38]. Melanin and related secondary metabolites are virulence factors in *C. lunatus* pathogenicity [7,8,38]. A closer inspection of the colonizing hyphae (Figure 2D) suggested that the dark-brown pigments might be an adaptive feature required to sustain *C. lunatus* virulence at higher temperature. This assumption is based on previous findings that proteins related to melanin synthesis and heat-stress responses are up-regulated in *C. lunatus *[7,8] during host colonization.

In this study, we found that *C. lunatus* invaded potato leaf in CDB medium. This suggested that CDB may not have negatively affected the interaction dynamics. Higher temperature-virulence phenomenon has been observed in *Cryptococcus neoformans* and *Aspergillus fumigatus *[39,40]. In these cross-kingdom fungi, loss of genes required for higher temperature growth resulted to attenuated virulence and at times death [39,40]. In our study, we found that conidia as well as hyphae at 28°C and 38°C were highly pigmented (Figure 2). Additionally, more necrotic zones were formed around the melaninated penetrating hyphae at 38°C (Figure 3). On the other hand, conidia were present and actively germinating, an important prerequisite for persistent virulence [9,12]. Put together, we speculated that *C. lunatus* concomitantly sporulates, discharges toxic-colored metabolites (Figure 1G) and produces diverse peptides during colonization of potato leaf (Figures 4, 5 and 6). Elsewhere, melaninated appressorial was reported in secretome analysis of *Colletotrichum higginisianum,* an important plant-pathogenic hemibiotroph [41]. Secreted secondary metabolites and candidate effectors can change the phenotypic characteristics of a host during interaction. Thus, this possibly explains why *C. lunatus* produces variable symptoms as earlier observed on different hosts [3,4,8-12].

In 2-D analysis, some of the spots were unique at 20°C with potato leaf and did not match with spots detected at 38°C with potato leaf where negligible change in pH was observed (Figures 5, 6 and 1B). Based on the changes in the secretome set (Figure 7), it is tempting to suggest that acidic microenvironment triggers *C. lunatus* to release more proteins. Also, we could not abandon the possibility that leaf protein underwent proteolysis during interaction. However, we failed to precipitate protein in treatments containing leaf without *C. lunatus* and also failed to find GPI-anchors on identified proteins. Based on these observations, we assumed that identified peptide spots were products of interaction secreted uniquely by *C. lunatus*. Nonetheless, the highest homology ranked peptide was CCO (spot# 38) secreted via classical pathway (Additional file 7: Figure S4 and Additional file 8: Figure S5). Adhesins are primary virulence factors used by pathogens to adhere to host prior to invasion [26,27] and only 10 peptide spots were found with adhesins motifs (Additional file 6: Table S2A, B). We concluded that majority of *C. lunatus* secreted proteins are highly soluble, positively charged (Table 1), discharged via the non-classical pathway and lack anchors to the cell surface.

Most phytopathogenic fungi progressively adapt to the genetic background of their host plants, thus creating a new type of virulent or physiological race [42]. This progressive process largely depends on the intensity of the pathogen-host specific interaction [43]. Nevertheless, during interaction, pathogenic fungi survived host counter attacks through coordinated secretion of their secretome weaponries primarily to harness nutrients and proliferate. Notably, the large number of up-regulated nucleic acid metabolic-related proteins, such as protein kinases (Figure 8: Table 1) indicated that under heat-stress or biological stress (i.e. with leaf), *C. lunatus* prioritized its proliferation. This is in agreement with the concept that protein profile of microorganisms change under biological or abiotic stress condition [44], which is also observed in this study (Figure 4). Intuitively, the abundant secretion of nucleic acid metabolic-related proteins enhances virulence as proliferation directly leads to large fungal biomass production. The secretome changes demonstrated that *C. lunatus* secretes varied repertoire of proteins depending on the ambient temperature (Figures 4 and 8). This is supported by unique unaligned peptide (spots# 27, 32, 34, 35, 36, 37 and 38) expressed only at 38°C (Figure 6: Additional file 6: Table S2A, B). This possibly could explain the survival and successful lifestyle of *C. lunatus* and related species at higher temperature [9-12]. Cautiously, the unique peptide spots could represent signature proteins required to sustain *C. lunatus* at elevated temperature only, not necessarily involved in pathogenicity sensu stricto.

Besides the peptide spots with known putative functions, abundantly expressed peptide spots with no catalytic domain and no predictable subcellular target got our notice. *C. lunatus* is a successful cross-kingdom pathogen and would cogently not over-secrete peptide spots# 6, 19 and 21 at 20°C with potato leaf (Figure 5: Table 1) and spot# 27 (Figure 6) at 38°C with potato leaf without a possible role in pathogenicity. As the normalization plot depicts (Additional file 5: Figure S3), *C. lunatus* secreted discrete extracellular weaponries at different temperature. Assuming that secreted proteins operates in a cooperative manner [37], it is reasonable to suggest that *C. lunatus* candidate effectors with no assignable functions exert an unforeseen synergistic role in temperature-dependent virulence. Secreted proteins with unknown functions is a common phenomenon in fungal-plant interactions [14,31,37,41]. In recent years, progress has been made in effectoromics [36,45-51]. Nonetheless, effectors are still ill-defined, but widely accepted that they manipulate host cell processes for the pathogen benefit [13-16].

Candidate effectors are understood to perform key functions prior to host colonization such as the provision of nutrients, mask recognition by the host defense system, cell-to-cell communication, detoxification of the environment, suppression of host-cell programmed cell death and killing of potential competitors [13,45-48]. Therefore, fungal secretome analysis is important. For instance, mining protein effectors that matches specific resistance-proteins can assist the deployment of engineered resistance-genes in crops and can also permit scientist to survey crops when a particular pathotype overcome the resistance-genes [47,48]. Importantly, effectors can be used as molecular probes to dissect focal immune responses, unravel the diversity of secretory vesicles and the cargo they transport [46]. We found that the secretome of *C. lunatus* on potato leaf shared motif- CLxxxxLHM (Figure 9). Cysteine is important and maintains effector stability in the extracellular space [36]. In addition, we observed a significant occurrence rate of CLxxxxLHM motif in other GenBank® fungal genome peptides (Figure 10). This shows that cross-kingdom pathogens share some common pathogenicity signatures in their secretome. For instance, in powdery mildew and rust fungi, motif Y/F/WxC [49] and *Oomycetes* effector motif RxLR-dEER [45,50] shared no positional conserved amino acid residues. Noteworthy, these pathogens colonize only plant hosts. Intriguingly, *C. lunatus* colonizes plant hosts [4-8] and animal hosts [1,2]. Critically, motif-CLxxxxLHM share features to motif-Y/F/WxC and motif-RxLR with conserved residue of cysteine (C) and leucine (L), respectively. In addition, histidine (H)-residue like arginine (R) is a basic amino acid residue and is rare in effector motif, but reported elsewhere [51]. Motifs with positional constraint amino acids such as M, L, H, N, Y, F, W and C have been reported in other fungi candidate effectors [49-52]. A closer inspection suggests histidine in motif-CLxxxxLHM is a unique signature in *C. lunatus* secretome on potato crop during invasion.

Pioneering effectoromics study indicated that fungal candidate effectors are most likely to be soluble and secreted into the extracellular matrix but do not form cross-link with fungal cell wall and show no homology to proteins with known functions [41]. Based on these criteria, we concluded that spots# 3 (*C. lunatus* genome peptide m118 v.2 accession no: 118437|e_gw1.13.184.1/ClEfc1), spot# 6 (19257|fgenesh1_pg.7_#_229/ClEfc2), spot# 19 (46252|estExt_Genemark1.C_210087/ClEfc3), spot# 21 (64448|estExt_fgenesh1_pg.C_160063/ClEfc4) and spot# 27 (132047|estExt_Genewise1.C_4_t40098/ClEfc5) (Table 1: Figure 11) with no homology to known proteins are candidate effectors of *C. lunatus* on potato crop. We found candidate effectors ClEfc1, ClEfc2, ClEfc3, ClEfc4 and ClEfc5 were secreted via non-classical pathway. As such, the candidate effectors did not have N-terminal signal peptide in agreement with conditions for non-conventional secretions [15,53]. Sophistication in *C. lunatus* invasion strategies is further enhanced by its ability to use the classical and non-classical secretory pathways to explore its microenvironment. Since only spot# 38 (Additional file 8: Figure S5) was identified with a TM-networking, this agrees with previous finding that many fungal effectors are soluble and do not often cross-linked with the fungal cell wall after secretion [41]. Our findings demonstrate that *C. lunatus* secretes majority of its effectors via non-classical pathway.

At 20°C with potato leaf, known virulence related proteins such as HSP70-related protein [7,41], tetratricopeptide (TPR)-related protein [54] and pleckstrin-like proteins were up-regulated. Based on the observation that the expression fold of HSP70-related proteins was greater than the cut-off limit, we concluded that it plays a decisive role in *C. lunatus* virulence on potato as previously reported in maize [7]. TPR-like proteins have a versatile helix associated with protein-protein interaction and intercellular trafficking [54] and a virulence domain [55]. Pleckstrin-like proteins controls diverse cellular trafficking interrelated with cell growth, survival, proliferation and metabolism [56]. In *Candida albicans,* pleckstrin-like proteins are understood to be involved in virulence and morphological switching [57]. Other putative proteins including protein kinases (spot# 1, 5 and 28), oxido-reductase (OXR, spot# 4) and cytochrome c oxidase (CCO, spot#38) were up-regulated. Although similar up-regulation was observed at the transcript level, their role in *C. lunatus*-increased temperature dependent-virulence is not clear (Figure 11A). Also, *C. lunatus* discharged an array of extracellular proteins that are often related with carbohydrate metabolism and proteolysis. Proteases play an important role in pathogenicity and maintenance of life cycle of fungi [37,46,58] and in this study, *subtilisin protease* (*Pte*) was constitutively expressed (Figure 11A). Toxin production in *C. lunatus* is believed to be positively correlated with up-regulation of *NTR *[7,8]. Furthermore, *SCD*, *SDR* and *NTR* are important genes for melanin biosynthetic pathway [7,8,41]. It was showed that up-regulation of *SCD* and *NTR* increased melanin pigments [7,8] and also improved the penetration strength of melanized appressorial [41]. Therefore, up-regulation of *SCD*, *SDR* and *NTR* might play important role in *C. lunatus* temperature-dependent virulence (Figure 11A). However, an evident up-regulation of inter-trafficking transcripts such as *WD40*, *Grlp* and *ALP* was observed (Figure 11A) which requires detailed investigation to decipher their possible role. Meanwhile, we suggest that *WD40*, *Grlp* and *ALP* may synergistically cooperate with candidate effectors *ClEfc1*, *ClEfc2*, *ClEfc3*, *ClEfc4* and *ClEfc5* to cause disease on potato.

## Conclusion

Classical 2-D is a powerful approach for direct analysis of secretome [16,37] and for the discovery of virulence factors in plant-pathogen interactions [7,8,58-61]. In this study, we have successfully used 2-D, MS-technology, RT-PCR and *in silico* tools to identify a set of 5 soluble candidate effectors of unknown function from *C. lunatus* secreted against potato crop at different temperature regimes. The findings throw light on the increased temperature-dependent virulence of *C. lunatus* in accordance with recent findings related to the release of pigments on maize and overexpression of stress related proteins [7]. Importantly, the discrete set of secretome provided new insight that can explain why *C. lunatus* can successfully thrives on a broad-spectrum of hosts under heat-stress conditions. The results showed that the sophisticated infection process of potato crop by *C. lunatus* comprises of: 1) intimate penetration of the leaf, 2) modulation of the ambient microenvironment pH, 3) release of colored secondary metabolites and 4) secretion of effectors candidate with diverse functions. In future, it will be interesting to integrate the analysis of secondary metabolites and the current secretome data to confirm the hypothesis generated in this study on *C. lunatus* thermo-pathogenicity.

## Abbreviations

1-D: One dimensional gel electrophoresis; Mr: Molecular mass; pI: Isoelectric pH; IEF: Isoelectric focusing; 2-D: Two dimensional gel electrophoresis; MALDI-TOF/TOF MS: Matrix assisted laser desorption/ionization-time of flight/time-of-flight mass spectrometry; CBR: Coomassie brilliant blue R250; CDB: Czapek Dox Broth; CDA: Czapek Dox Agar; IPGs: Immobilized pH gradient strips.

## Competing interests

The authors declare that they have no competing interests.

## Author’s contributions

BL conceived the experiment, run 2-D electrophoresis, performed *in silico* analysis, analyze gels and wrote the manuscript. SDW performed the fungal-potato interaction and assist to optimize the culture conditions. PR had the main responsibility for studies involving electrophoresis and interpretation of MS spectral, microscopy and proofreading of the manuscript. PKB assisted in gene expression studies. MWS assisted in statistical analyses. SG assisted in harvesting the extracellular proteins. CKS assisted in microscopy studies. NCT supplied all the reagents and assisted in microscopy analysis. All authors read and approved the final manuscript.

## Supplementary Material

Additional file 1 **(Fasta): Protein sequences of *Cochliobolus lunatus *** **m118 v.2 genome peptides matching with tagged peptide spots on gels used for motif searches.** Click here for file

Additional file 2: Table S1 Details of primers sets used in semi-quantitative RT-PCR. Click here for file

Additional file 3: Figure S1 A 2-D secretome map of *C. lunatus* in CDB cultured at 20°C without potato leaf*.* Identified spots are indicated with arrows. The immobilized pH gradient scale and standard molecular weight scale are shown. Click here for file

Additional file 4: Figure S2 A 2-D secretome map of *C. lunatus* interacting with potato leaf in CDB medium cultured at 28°C*.* The immobilized pH gradient scale and standard molecular weight scale are shown. Click here for file

Additional file 5: Figure S3 Dispersion of positional constraint normalized spot volume plots generated in Progenesis SameSpot 4.1 suite. The graphs depict the distribution of spots against a reference gel for *C. lunatus* secretome (in this case, Figure 4). (A) Secretome plot at 20°C with leaf and normalization factor is 2.1. (B) Secretome plot at 38°C with leaf and normalization factor is 1.38. (C) Reference gel plot obtained here is considered as 20°C with leaf use for normalizing the alignment and the normalization factor is zero. Click here for file

Additional file 6: Table S2 Details of peptide spots excised from 2-D gels. (A) Identified proteins that match unique protein models predicted from *C. lunatus* m118 v.2 genome peptides. (B) Details of identified proteins that did not match unique protein models predicted from *C. lunatus* m118 v.2 genome peptides. Click here for file

Additional file 7: Figure S4 Ten best peptide spots hit maps corresponding to unique protein models predicted from *C. lunatus* m118 v.2 genome peptides available at *http://genome.jgi.doe.gov* with E-values ≤10^-5^. Open reading frame map are indicated with broken arrows in the forward (left to right) direction with each hit associated with the protein accession number and protein score. Click here for file

Additional file 8: Figure S5 Signal peptide and transmembrane network prediction profile for spot# 38 in SignalP4.1 [22]. Spot# 38 (jgi_Coclus-46026) was identified as cytochrome C oxidase. Discriminatory cut-off value D = 0.5 indicates the presence of signal peptide with transmembrane network. Click here for file

Additional file 9: Table S3 Details of motifs hit scores generated in MAST [29] and FIMO [30]. The likelihood scores of motifs in 23 fungal genome peptides and data on the strength of motif CLxxxxLHM tested against 23 fungal genomes at *P* < 1e^-4^ are depicted. Click here for file
