# Erratum: Secretome weaponries of *Cochliobolus lunatus* interacting with potato leaf at different temperature regimes reveal a CL[xxxx]LHM - motif

**DOI:** 10.1186/s12864-015-1337-3

**Published:** 2015-05-01

**Authors:** Bengyella Louis, Sayanika Devi Waikhom, Pranab Roy, Pardeep Kumar Bhardwaj, Mohendro Wakambam Singh, Sailendra Goyari, Chandradev K Sharma, Narayan Chandra Talukdar

**Affiliations:** Institute of Bioresources and Sustainable Development (IBSD), Takyelpat, Imphal, 795001 Manipur India; Department of Biotechnology, The University of Burdwan, Golapbag More, Burdwan, 713104 West Bengal India; Department of Biochemistry, University of Yaoundé I, Yaoundé-BP812 Yaoundé, Cameroon; Department of Biotechnology, Haldia Institute of Technology, Haldia, 721657 West Bengal India; Regional Centre of the Institute of Bioresources and Sustainable Development (RCIBSD), Gangtok, 737102 Sikkim India; Department of Biotechnology, Guwahati University, Guwahati, 781 014 Assam India

**Erratum to**: *BMC Genomics* 2014, **15**:213 doi:10.1186/1471-2164-15-213

The unpublished genome data for Cochliobolus lunatus m118 (PIs: Nada Kraševec, B. Gillian Turgeon) were produced by the US Department of Energy Joint Genome Institute under Contract No. DE-AC02-05CH11231.

