# Supplementary material for: Secretome weaponries of Cochliobolus lunatus interacting with potato leaf at different temperature regimes reveal a CL[xxxx]LHM - motif
Source: BMC Genomics. 2014 Mar 20;15:213. doi: 10.1186/1471-2164-15-213 (PMC4000054; doi:10.1186/1471-2164-15-213)
Supplement: Additional file 1 — (Fasta): Protein sequences of Cochliobolus lunatus m118 v.2 genome peptides matching with tagged peptide spots on gels used for motif searches. [file 1471-2164-15-213-S1.docx]

>jgi_Coclu2_123093: spot 1

MVQASEVLSRKTGVIVGDDIHKLFKHAQSEGYAIPAINVTSSSTVVAALEAARDAKSPIILQLSNGGAAYFAGKGVSNSNQEASIAGSIAAAHYIRSIAPAYGIPVVLHTDHCAKKLLPWLDGMMDADEAYFKQHGEALFSSHMIDLSEEPKEWNIATTKKYLQRAAPIKQMIEMEIGITGGEEDGVNNEDVDNNSLYTQPEDIYDIYKELSEVSPLFSIAAGFGNVHGVYKPGNVKLRPELLQKHQAYVKEKTGAKDDKPVFLVFHGGSGSSVDDFRQAISYGVVKVNLDTDMQWAYLSGIRDYIQSKSGYLQTQVGNPDGDDKPNKKYYDPRVWVREGEKTMSQRIKVALDDFYTAGKA

>jgi_Coclu2_16455: spot 2

MTLSSPLPSFSSPASSRLPAARRASFSGQLAFLVHSQETVANHMPPDVDNKALARQKRRRTSKEDEDILKSEYLKNPKPSKAARLEIVKKVALGEKEVQIWFQNKRQNDRRRSRPLEPSSTASFLSSSSAMSDPLTEDEVMPGARRIDNAPEPKESDECLKSDPPEQPATPELTSDETIPEPRTIDTALSVAAESERANETAVQPSTEAQAETAPVQTTDSQQAVSSSQGATQSRTSWISNRRSASFVRYLEDYTPEVITFPNAPPRPSESPETTAKTPSRPLKRTHSFMRLSTNENGTARIVTDLDKTPSPPKSKKTPSSFSRTAAGLRRSYSAAGLNDRLAAAARGEPTPKIPRTVSSIGRSRDSRAWEFWCDPETRSTTSLTARAEQEESGSAADAIGILRANRRILARNQARQNSSPLTGRHSLHKVLGTPSVKKSRGPMQRASTISGRLSHNDYSDYKKGGDSTESDEFPQTESDKENWEPNAPKTVCRDRQVAATPPASRGARQILGENRELMSQSSSLGALLAKEKRSGGKRVIDPEQDDELRQFMNGDGASGRSSINSAEEAGCVEGLLKLSQGQWR

>jgi_Coclu2_118437: spot 3

MPPKRTHNADEAQLSLKDKLQNLQGSNARGGRRNGGANVMNGSGLKEVDNASTNSGHTSVDLSSSGNIKWSSQDTSVLHGYRRAYRLDCPSSFKNPLSHVVLNQGIGRMSPTMARPKSKRRVHKDQLGLAVKKSFNSQAVTESDVIVDWLYKSKHQDKEFRVRFAPYRK

>jgi_Coclu2_125544: spot 4

MAQEFKLKDVTSLQMKNGEKKEAEVEGVEGGKVLLLKVQDQVHATSPNCTHYGAPLVKGVLTPEGRLTCPWHGACFKVSTGDVEDAPALDPIAKYEVIEKDGGVYVKTTEEALKANRRHLNIKCSSVSEDKVLVIGGGSGTLGAIEGLRGGGYTGKITVISKEGYQPIDRTKLSKALLADISKLAWRSKDFYKDGSIDIIEDEAKSVDFSGKKVSTKSGKEYEYTKLVLATGGTPRWLPLEGLKGDLGNVFLLRTLPDAQNILKAVGDNGKKIVVVGSSFIGMEVGNCLAGMKNDVTIIGMEEEPMERVMGKKVGAIFRGLLEKNGVKFKMGASVDKATPSKSDSSKVGAVHLKDGTALEADVVIEGVGVAPATEYLKGNSSITLEKDGSLKTDESFAVQGLSDVYAIGDIATYPYHGPGGNGSPVRIEHWNVAQNAGRSVAHIINNPGSKPKPFIPVFWSALGSQLRYCGNTVGGYDDVFLQGEPEKASFVAYYTRGETVVAVASMMKDPYMTQAAELMRRNKMPSKSELQKGVDILEISLPSEVKI

>jgi_Coclu2_24606: spot 5

MAPPRTSSHRSTSNGAPSQSHHRDESSQSRRRAKESPRQQGTTSTRSQPTHSRSATAIGQPPVNTSLPREESAVINRIVVSDTNEDIARAQARQGEAVPVHAAGANITPITGLSLVGSEGVDDGGRGAGGKSRQDHSKSENAKRSKFGNYILGQTLGEGEFGKVKMGWKRDSPIEVAIKLIRRETLGSNSNRLQKIYREIHILRGLDHPNIVRLHEMVETERHIGIILEYASGGELFDYILNHRYLKDGPARKLFSQLISGVGYLHRKGIVHRDLKLENLLLDRNKNIIITDFGFANTFDPNDELSEEIEYRLGDKDFMKSLGLDGSDATRRGDLMQTSCGSPCYAAPELVVSDSLYTGRKVDVWSCGVILYAMLAGYLPFDDDPANPEGDNINLLYKYIVSTPLTFPEYVTPHARDLLKRILVPDPRKRADLFEVARHSWLADYAHVVGFITSSNTNVAEAQQASVYSKDTFEPQPALARSASVREPAKPHIASAHGGLQTKRDAINPTAEKPKTTRDTKRRTVQVEYVAPSSQTARGEASPPAETAPKVRGKEAEVAAPITDAHQPAIGARPSRSSGAVQAPARKQEPQRSTSEYTGFANAPTSATRPSTSGALGSRLPSRGNSYGQPSVATVAQTNAEGRFSQPKGKQYSISAPYAQHDASEAAEPPNIGQPSAHRAQSIQMGPHGPKGTHRRSNTVTETLGRMTSMFSGRQPSYQDPKASLTSQGSYGYPDDKKQRNYPPTSMTGPMSNDNVSATTGPRPSMESSRRTSFGFSRKNNSSSNSNNGDGTKPSRRFSLIPSSLSRTFTSRESAPSQSSHERRASSSRPRASSRPGGMSFGRGGLSRSPSQSTQGSNMPGAYDGHQDRLRNMHMNAAGPSSAPPNQTHFNSYNQSAAEFDEKFPNPQEAHPAAQAGRPHQQIADDSDASETHGPGRISISQQFGQRNTSNPYPPGMGGDEFQQQQRKGVLQKSRRFADAYDEQGSANKGSSGSSKKVMDFFRRIGKQRTSSR

>jgi_Coclu2_19257: spot 6

MDTFRFRLNCIDYYQAPPTDLDPLLRRNPGPLSRLGAPEVSVIRVFGATDTGQKVCAHIHGALPYLYVEYNGSLETDVVNSYILELRTSIDHALATAYRRNTHDTKSIYVGHISLVKGVPFFGYHVGYKVFLKIYLLNPMHMTRFADLLHQGAILNRVFQPYESHLQYLLQWMCDYNLYGCDYIDCAKVQFRGPVPDSDELDTTIHKWHDASIPDEFMADEDQYPRQSHCTLEVDICVQDILNRHQLQYRPIHHDFLERSSQTLQNPDDKFVPSMAGLWRDETRRRKLRMGLTSPSSSPFPAEVLVSMSADTRDTRKGGWIHEDEYREIVSSLIEEEKRGKDDVSFDTFTKPAEGQHHIKTAAESVEDLYPEVLSTQRLPDHNLEDESETSEEDSAHFLGGADLMSDDLDPLPQYQHGGVILGDDDGRKQADADNQSDDEEHSMDPLTLSLLQGADEAAGNDAHIDEGIDLVDPSDEDELPNISSPSTMRKRRTESTDEDYNKRQRILPEAQGTGRRVSFRNALNEGNNQTASSAQRSPWEEPPPAGQLSQASIKAQGLGGPTTLSFPVVKNPNAPETLHRLSQSGYSNSQEGPKTPGTKQFNVRASHRSPVTPWTHQKASPAIPASSTPTARSEATSLVRKLSESFRHYRPTVYYGYLPPTAEVVESTMMHAGLPPVIYQDAYYSDEKDVPDRPREYAGREFKLQSTTLPYLPPFDPAGASLANIGEYPPIVVDKREQAATYEARSKRCGLRHWEFSKPAPSFEEVREWTLCEARRPKTGKLDTKTKHLPKPTIRRSDPVSHLSQIEGPTQKNPHGFKYSQKQKDTSVKHESQYMSIMSLEVHVNSRGSLAPDPAEDEIQCIFWSVQGEENAVVDRPRTGILCFSEEDGIAVRIAKQVPVEVEYEEDELDLINRIVDIVRQFDPDILTGYEVHNSSWGYLIERARMKYEYNLCDELSRMKSQSNGRFGKEADRWGFTHTSTIRITGRHMINIWRAMRGELNLLQYTMENVVFHLLHKRIPHYQHSDLTTWYRNGKPRELSKVLNYFLTRVQLNLDLLDANDIVPRTSEQARLLGVDFFAVISRGSQFKVESTMFRIAKPENFILVSPSRKQVGQQNALECLPLVMEPQSAFYSSPVLVLDFQSLYPSVMIAYNYCYSTCLGRIVSWRGRNKMGFMDFKREPQLLELVKDYINIAPNGMMYVKPEMRKSLLAKMLGEILETRVMVKSGMKEDKDDHTLQKLLNNRQLALKLLANVTYGYTSASFSGRMPCSEIADSIVQSGRETLEKAIAIIHETEKWGAEVVYGDTDSLFIHLKGRTKDQAFTIGEEIAAAITAANPRPIKLKFEKVYHPCVLLAKKRYVGFKYEYRNQKEPVFDAKGIETVRRDGTPAEQKIEEKALKLLFRTADLSQVKRYFQEQCAKIMEGRVSIQDFLFAKEVKLGTYSNKGHAPPGALIATKRMLADPRAEPQYGERVPYVVITGAPGSRLSDRCVSPETLLQSDHLELDAEYYISKNLIPPLERIFNLVGANVRQWYDEMPKVQRIRNISIPIAPKYDNNGNVLVGGSNNSLKRTLESYMKSSNCLVCRSKLGPPASAYSSDDAYAMLPLCSKCIRRPAHSLLALKYRIWSSETRIAEVDTVCRRCSNLAWNEEIRCDNRDCPVFYTRTKEKSRLAALRDNLEPVVDIIQALEEANEERVVEHEGEVGRRSNDDELRW

>jgi_Coclu2_115068: spot 7

MSSSSDREPSRDRELVRHSHSFSSDRMIPMWDSADPERAPPPLPLNPGSPNLTTRPNTSAAIMQAAKALEEKARESLPASSYTTNPMPLKSLSPERALIKGNQHKRMQSLQPGSVRDLRSYLDNRSPDRSYLDRSPERSPDRDRPSSRGGRPISRDWEKDYFGKDADASPTRHVTPSPTTRFEIKDTPALRPSHRPFLGENTPPSATMMALQTMSIPQHMLDPPPSTLTKEARSPPPAAASPPAPAPASATAPLQGTVPPQSFENIASQLVSINNIVSSLQKDMSQLSRRSKDNATDLVSLKEATNLRDEDIRKSLKDLLATMSQKQEAAAMNGDASRSSSLNLQDPHMTPTKQFTFPRMASTSPWADERIGSPNPYSVEGAASVAMLEKIIREMVTKDGQERLVANLQKIVDKATGETAQKVTELVEFVKQGSLGGSIHNAVPGSAGFQPSPGSGALTRTISADSKVYSSPKAADFVSEEMLKFLRKIKDSVAESGCVTMATKTLIQDLRGEVLGMGRELARKLEEAEQARTVESDSGSSPSNEQINTILQEGLANLKEHMDKVMRERRRQSMSSVVTRNTVDNNEVYDVVKQALAERGLDQPSAQSASLDQEAIISAVREAFDGVEMNPNREVQQVGLERDEILQCLREGLQDFTGSGAVTKEEIETMLHDSLQQIQLPPPINEVHEIREEVLMAVRESLEELKPALAAQQPPPAESLSRELLEEIIRQALAEHNSDGLEIEIPPESIQDAVKAATEAHNESVMQRLQSMVEEMHTAFEGYSSSSGKDTEQVLTTLREGLDSLRTEIQASSSQPREITQDGEMIDQFKAELDKLREDVQGYVAEGTRGDQEWSREDMVTYLKSEFESLQEKLSSQLLPPSKSNEEILAALNAGFEEMKSQVALRSADSDDVDSNEDISEAIKHEFDQLKEVVLGDTSSNKNDILEKLQAGFDGLHTLMAEKQTASTSNDDMLASLKEEFEQLKETLGGSLVKSDSSADKDEVVNAVRELIEGLHSSQETTSKENLAHIRSELEALKDSLSNALVPSGENDKSEMMMVVKNALDEIKSSLTSAGVNEELLEAFRGELDHLRQSNGLIRQHSRADTEEVLEAVKLGLDDLQSHMDKKFNTPERNTTATDDIIDAMNEGLESLRADVSKIVEKPIDMSVSYEILDTLKEGLAALRADVEMLKGVRHEAELAPEDIPLPAGNEVALVENPESEEITSREISEDMSEPDLPPQPVAQSLDVAKMERLLTQIQDKVQAMDATAKEPAPPSEPAAPAGAVMKEDLGVIEELLKDVQAAVLLMQDRETSPPELEGLAKKEDTDAIETLLANTKAKIEELVLPDPATAVTKEHLEDVELVVRTTSETLEALSKKFEEEGATKADVTVVQVLADDIKVALDEMKAAKPEDESNPHATKADIDAVTLLVDDLKVKLDDLKIPDQEELASKAELEQLTGLIHDFRDSHEKMKDTYEADIQKTANNFDERRAEATQIVEDIAGVKAALDEMREEIKTSLIEGGPIESLQASFKTMEETIGANNVTADVKELMEIVTREFERAHGSIEGLQNDNAEKSALQLEKHDETKAAVIAAFTESLEDKFNTLMAKYDDAQLLADELAKVMKEKSEEQEKMLESTKATADELRLTIDTLGATIAGMNDRFEGATTQWSTDSTTVLSKVDETLTKFDEQKLATMEKLEEQKLETIAKLEEHIAKFEELKLDDKTEHSHTRDEIKNVEAIFNNLQDNITEYHPKFMVALHEIEALVKAHYEHAQKCKEEADENARVWNEQAKARSDELQAHFENLPKLLPAPAESESKYDDGPVQEKLDKLLAIEPAPSYDDSPVHQKLDKLLGHADDATRAAANAEKLDEIHNQVKATAAELSEFVAKQTQFITDGNEAKEREAEELLLLIERRTTQKEQLEIDLESLRAEKANQKEQLMADIQEMKAEKERVVQELKEEKERVMTELQNEKDLTMLELKEEKDALLAAVAALQAERENLANQKVRLTGEVSSLQTALEIRREELHIMDTKADALERRILNGIMDHSRALMMTKGGSKNPSKSKKRVSINPDAEGTRGTQSPSAAANGLSLALKPRAAPRKNGQPVNPTQRRILSLSQISGNTPTGAQAYPTTTASANANAGLKRSHSVKTSNYSRKGSWGGKPSTSIANKENGALPEVDENEAMAPVSHDIIEEDVQSETGTERRYSVDDYPESYAEGETPGYDGRSSFGGEGSEYTYASGTSYMTGSDIDHDRRTSYGAQSVHSAARGEEPTNEGSEGSEYSDDEEDNERTAIIDPSEVNSSVVTTATADSEIDLPTQSEIVRAVDAVKAEMKDHYSPPSDSGVGTDLATVPLEHEAEHDADYFRRAAEEEASVVG

>jgi_Coclu2_129342: spot 8

MGAIKRKAEQGATPSKKAKGASNDRSAKRRKSDVDQQSPAKAKLESAAPASVFKDEEKKAFPRGGASVLTPLEHKQIQIKANQDVLFEQSGQKRPGGDDGEGYSDMGSEDDATKTAPKTRKKPLKKSKKTHESGDKEDVVKVESLSYKITDISHQDLVLALPNNLVGYVPLTAVSDKLNERLEKLLKEDENDKEDGSDEESFEDVDLKDLFSVGQYLRACIVATSGEGAKARKRLELSIEPKLVNAGLTKRKIPVNGMIQASVVSNEDHGLVMDLGLNDTTLKGFLPKSELGPNVQHSKVQEGTVFMCLVTGLNSDGRIVKLSADHTKAGNLTKGNTLTDAPTIDVFLPGTAVDLLVTETTPNTITGKILGLIDATADAYHSGATERATDISQKHKIGSKVKARILFTCPGSDPRKVGVSLMDHVLALSTRMSGKPKERKAPTDILPISTIVESAKVVKVAQNQGAFFDLGIKDVIGFAHISRLSDDKVDILSEEAGAFKLDSQHKARVVAYNAIDGLFQLSLEKKVLDQPFLRIEDIKAGEIVKGKVHKLIADKTGATAVLVNLAEGITGLVPEMHLADVRLQHPERKFREGVPVTARVLYTEPARHQIQLTLKKSLVNSDVKPWTNYEMLSEGAAGPGILVSVRRNGATVRFYGNVKAWLPVAEMSEAFIDDATRHFHAGQVVNVRVLAVDAKERQLLVSCKDPAAVDTNKEAAFNALNPGGIVKGTVVEKSEESATLDIGNGVKGILRLGHLTDGSEKKDISTMKKIRVGGTLEDLVVLTKHGKSKTATVSNKPSLRKDAQASKLAISVEDLQAGETVHGFVRGILSDKVFVELGNGISGALFKSQMPEEMASMPDFGLRKDQSVTARVTHVDTGKGFFWLSMKAESDADAAKTKAVTPAKAGEALVNPVDPKIISTDDIKFGTLMTVRIRSVKDTQLNVEVAANVPGRISVSELFDSWDSIPDKKRPTAHFKMNDTIQARVLGRHDPRNFRFLPITSRSSNKTTTYEMTAKTEQIKTEADVLSLEKITTGSSHIAFINNIADRHVWVNISANIRGRIDFFDLTDDLEKLSDVEKNFPVGSALKVRVKAVDAANGKLDLTAASTVTGKTLSLQDLKVGHVLPARVTKLHDASIVVQINESIAAPIFMEQLADDYDKAQPKNFMVGDVLRVCVISIDIPNKKLGLSARPSRVLSSSLPIKDPEITDRAQLKVNQVVRGFIKHIANNGVYVRLGPNVEAYVRVSHLSDEYIKDWKTAFHVDQLVTGKVISNKEDQRNPQLSLKTSVIKEDYVEPLEFGDLKVGQIVTAKVRHVEDFGVFLVVDNSNNVSGLCHISQIADAVVDKEKVKEMYKKDDAVKAKVIKIDPKSRKVSFTLKYSQVKGEGEDEEMQNASDVESSEDGDSDGGIEIDDDTDMRSVKSAESDDDIELADADDSEDDSDKDEPKATAQGLSTSGFDWTGATLDADQQKDAAESESEDDAPKKKKKSKKATIKEDRTGDLDAYGPQSVADYERLLLGQPNSAELWVRYMVFQRELNEIEKARQIARRALATINPREEKEKLDVWTALLHLENDFASDDTINSVFKEACQNNDAREVHERMIKIYISSGKIDKADELYQSMTKNKSFTPDPKFWLSYATFLMDVVQPPSPARARALLQRATQSVAAPQHRYLTQKFAALEFKSANGDIERGRTIFEGLVDTFSKKGDVWDMYLMLEQSHGEPDKVRDLFERMTKVGKASRVRGVFKKWAEWESSVGNKKGVDRVKKLEEEWRQKRAENDNE

>jgi_Coclu2_115068: spot 9

MSSSSDREPSRDRELVRHSHSFSSDRMIPMWDSADPERAPPPLPLNPGSPNLTTRPNTSAAIMQAAKALEEKARESLPASSYTTNPMPLKSLSPERALIKGNQHKRMQSLQPGSVRDLRSYLDNRSPDRSYLDRSPERSPDRDRPSSRGGRPISRDWEKDYFGKDADASPTRHVTPSPTTRFEIKDTPALRPSHRPFLGENTPPSATMMALQTMSIPQHMLDPPPSTLTKEARSPPPAAASPPAPAPASATAPLQGTVPPQSFENIASQLVSINNIVSSLQKDMSQLSRRSKDNATDLVSLKEATNLRDEDIRKSLKDLLATMSQKQEAAAMNGDASRSSSLNLQDPHMTPTKQFTFPRMASTSPWADERIGSPNPYSVEGAASVAMLEKIIREMVTKDGQERLVANLQKIVDKATGETAQKVTELVEFVKQGSLGGSIHNAVPGSAGFQPSPGSGALTRTISADSKVYSSPKAADFVSEEMLKFLRKIKDSVAESGCVTMATKTLIQDLRGEVLGMGRELARKLEEAEQARTVESDSGSSPSNEQINTILQEGLANLKEHMDKVMRERRRQSMSSVVTRNTVDNNEVYDVVKQALAERGLDQPSAQSASLDQEAIISAVREAFDGVEMNPNREVQQVGLERDEILQCLREGLQDFTGSGAVTKEEIETMLHDSLQQIQLPPPINEVHEIREEVLMAVRESLEELKPALAAQQPPPAESLSRELLEEIIRQALAEHNSDGLEIEIPPESIQDAVKAATEAHNESVMQRLQSMVEEMHTAFEGYSSSSGKDTEQVLTTLREGLDSLRTEIQASSSQPREITQDGEMIDQFKAELDKLREDVQGYVAEGTRGDQEWSREDMVTYLKSEFESLQEKLSSQLLPPSKSNEEILAALNAGFEEMKSQVALRSADSDDVDSNEDISEAIKHEFDQLKEVVLGDTSSNKNDILEKLQAGFDGLHTLMAEKQTASTSNDDMLASLKEEFEQLKETLGGSLVKSDSSADKDEVVNAVRELIEGLHSSQETTSKENLAHIRSELEALKDSLSNALVPSGENDKSEMMMVVKNALDEIKSSLTSAGVNEELLEAFRGELDHLRQSNGLIRQHSRADTEEVLEAVKLGLDDLQSHMDKKFNTPERNTTATDDIIDAMNEGLESLRADVSKIVEKPIDMSVSYEILDTLKEGLAALRADVEMLKGVRHEAELAPEDIPLPAGNEVALVENPESEEITSREISEDMSEPDLPPQPVAQSLDVAKMERLLTQIQDKVQAMDATAKEPAPPSEPAAPAGAVMKEDLGVIEELLKDVQAAVLLMQDRETSPPELEGLAKKEDTDAIETLLANTKAKIEELVLPDPATAVTKEHLEDVELVVRTTSETLEALSKKFEEEGATKADVTVVQVLADDIKVALDEMKAAKPEDESNPHATKADIDAVTLLVDDLKVKLDDLKIPDQEELASKAELEQLTGLIHDFRDSHEKMKDTYEADIQKTANNFDERRAEATQIVEDIAGVKAALDEMREEIKTSLIEGGPIESLQASFKTMEETIGANNVTADVKELMEIVTREFERAHGSIEGLQNDNAEKSALQLEKHDETKAAVIAAFTESLEDKFNTLMAKYDDAQLLADELAKVMKEKSEEQEKMLESTKATADELRLTIDTLGATIAGMNDRFEGATTQWSTDSTTVLSKVDETLTKFDEQKLATMEKLEEQKLETIAKLEEHIAKFEELKLDDKTEHSHTRDEIKNVEAIFNNLQDNITEYHPKFMVALHEIEALVKAHYEHAQKCKEEADENARVWNEQAKARSDELQAHFENLPKLLPAPAESESKYDDGPVQEKLDKLLAIEPAPSYDDSPVHQKLDKLLGHADDATRAAANAEKLDEIHNQVKATAAELSEFVAKQTQFITDGNEAKEREAEELLLLIERRTTQKEQLEIDLESLRAEKANQKEQLMADIQEMKAEKERVVQELKEEKERVMTELQNEKDLTMLELKEEKDALLAAVAALQAERENLANQKVRLTGEVSSLQTALEIRREELHIMDTKADALERRILNGIMDHSRALMMTKGGSKNPSKSKKRVSINPDAEGTRGTQSPSAAANGLSLALKPRAAPRKNGQPVNPTQRRILSLSQISGNTPTGAQAYPTTTASANANAGLKRSHSVKTSNYSRKGSWGGKPSTSIANKENGALPEVDENEAMAPVSHDIIEEDVQSETGTERRYSVDDYPESYAEGETPGYDGRSSFGGEGSEYTYASGTSYMTGSDIDHDRRTSYGAQSVHSAARGEEPTNEGSEGSEYSDDEEDNERTAIIDPSEVNSSVVTTATADSEIDLPTQSEIVRAVDAVKAEMKDHYSPPSDSGVGTDLATVPLEHEAEHDADYFRRAAEEEASVVG

>jgi_Coclu2_129342: spot 10

MGAIKRKAEQGATPSKKAKGASNDRSAKRRKSDVDQQSPAKAKLESAAPASVFKDEEKKAFPRGGASVLTPLEHKQIQIKANQDVLFEQSGQKRPGGDDGEGYSDMGSEDDATKTAPKTRKKPLKKSKKTHESGDKEDVVKVESLSYKITDISHQDLVLALPNNLVGYVPLTAVSDKLNERLEKLLKEDENDKEDGSDEESFEDVDLKDLFSVGQYLRACIVATSGEGAKARKRLELSIEPKLVNAGLTKRKIPVNGMIQASVVSNEDHGLVMDLGLNDTTLKGFLPKSELGPNVQHSKVQEGTVFMCLVTGLNSDGRIVKLSADHTKAGNLTKGNTLTDAPTIDVFLPGTAVDLLVTETTPNTITGKILGLIDATADAYHSGATERATDISQKHKIGSKVKARILFTCPGSDPRKVGVSLMDHVLALSTRMSGKPKERKAPTDILPISTIVESAKVVKVAQNQGAFFDLGIKDVIGFAHISRLSDDKVDILSEEAGAFKLDSQHKARVVAYNAIDGLFQLSLEKKVLDQPFLRIEDIKAGEIVKGKVHKLIADKTGATAVLVNLAEGITGLVPEMHLADVRLQHPERKFREGVPVTARVLYTEPARHQIQLTLKKSLVNSDVKPWTNYEMLSEGAAGPGILVSVRRNGATVRFYGNVKAWLPVAEMSEAFIDDATRHFHAGQVVNVRVLAVDAKERQLLVSCKDPAAVDTNKEAAFNALNPGGIVKGTVVEKSEESATLDIGNGVKGILRLGHLTDGSEKKDISTMKKIRVGGTLEDLVVLTKHGKSKTATVSNKPSLRKDAQASKLAISVEDLQAGETVHGFVRGILSDKVFVELGNGISGALFKSQMPEEMASMPDFGLRKDQSVTARVTHVDTGKGFFWLSMKAESDADAAKTKAVTPAKAGEALVNPVDPKIISTDDIKFGTLMTVRIRSVKDTQLNVEVAANVPGRISVSELFDSWDSIPDKKRPTAHFKMNDTIQARVLGRHDPRNFRFLPITSRSSNKTTTYEMTAKTEQIKTEADVLSLEKITTGSSHIAFINNIADRHVWVNISANIRGRIDFFDLTDDLEKLSDVEKNFPVGSALKVRVKAVDAANGKLDLTAASTVTGKTLSLQDLKVGHVLPARVTKLHDASIVVQINESIAAPIFMEQLADDYDKAQPKNFMVGDVLRVCVISIDIPNKKLGLSARPSRVLSSSLPIKDPEITDRAQLKVNQVVRGFIKHIANNGVYVRLGPNVEAYVRVSHLSDEYIKDWKTAFHVDQLVTGKVISNKEDQRNPQLSLKTSVIKEDYVEPLEFGDLKVGQIVTAKVRHVEDFGVFLVVDNSNNVSGLCHISQIADAVVDKEKVKEMYKKDDAVKAKVIKIDPKSRKVSFTLKYSQVKGEGEDEEMQNASDVESSEDGDSDGGIEIDDDTDMRSVKSAESDDDIELADADDSEDDSDKDEPKATAQGLSTSGFDWTGATLDADQQKDAAESESEDDAPKKKKKSKKATIKEDRTGDLDAYGPQSVADYERLLLGQPNSAELWVRYMVFQRELNEIEKARQIARRALATINPREEKEKLDVWTALLHLENDFASDDTINSVFKEACQNNDAREVHERMIKIYISSGKIDKADELYQSMTKNKSFTPDPKFWLSYATFLMDVVQPPSPARARALLQRATQSVAAPQHRYLTQKFAALEFKSANGDIERGRTIFEGLVDTFSKKGDVWDMYLMLEQSHGEPDKVRDLFERMTKVGKASRVRGVFKKWAEWESSVGNKKGVDRVKKLEEEWRQKRAENDNE

>jgi_Coclu2_19962: spot 11

MAPPQTPKNKKQTQNASTPHITDSMGPASAVGDMPPPLVSQPLETYEKAQEDEREVLKAVFMDDYEESEAKGAWSKTTDRVLRLKLTSFSDSNIFVTLRAKLTATYPKSLPELTLEDCSSLRKTTWDKLYGLLKRRPQELVGEVMIYDIATAIQEILEDEIAVRETDGAYANLDAERAMQEAAAAEQLRHQEAELQRKRDEEKAQEERLLQQMVGDEIRRKDLMAKRKKNRSSVMTPPPDFPSGDSVNYISFDRTTSIQYEDTTIQCSAVENPLPFRSGPLTEQLLVKPMGTPVALTLVLKRARVGDENGDTDPALKKAIMAFEDEMEEVKRLRQSAVMTVLDFKIEHLPESGWEISILMEHADRGSLGEKLADDGPLQVARVRSWTIELLEALDFYHRNGIVHKRVHPNNILLKRSSGGGITVHLADASFQDSLYRLQSLSKDSRPLKSQRSFYWIAPELSQDLQHTRKTDVWDLGVVFLQMLFGVDAPDKYNSPKDLSDILGCSEPLQEVLRKFFKPDPKKRHSAFDLIPSEFLRDDVDVYDRPRTPVRSRNSSTSLGRYRLRRESSSGMVPTYSRYANDWVEQGRLGKGGYGEVVKARNKVDGRLYAIKKIKQKSAAALSEVLSEVMLLSRLNHSCVVRYYTAWPEAEADYDEDSGSSDGDESDWSSDVSPTNTDGEIHGKSTGGLDFISSSGYPKIEFGSDAESEDDVGAVIFGSDSEDDTGSASIGHSPPGLKKRAMSDSNPTKSRSILYIQMEFCEKQTLRDLIRGGLYDDPDEYWRLFRQILEGLAHIHGHGIIHRDLKPDNVFIDLAKIPKIGDFGLATSGQYQRPDTRSSNDNQNGGDMTRSVGTALYVAPELSSSVTGDYNNKVDMYSMGIIFFEMCFPLRTAMERDKVIRSLRERKHTLPPEFETPEKALQGGIIESLISHRPSERPGCTELLRSGKVPVQMEDEAVKEALKALSDRNSPHYAKMMAALFSQKPDTQARDYAWDLSTANQTIKANDVLLQNLVRDRLAMIFRSHGAVEIQRQVLLPWSDHYANSNVVKLFDPSGTLVQLPYDLTLPYARSIGRGGAFLEKTFTIGHVYRENYTGSAPRSNGEADFDIVSYDAIDLALKESEVLKVVDEIIDGFPSFRSTPMCFHLNHADLLEIVMEFCRIPAPKRTAVKAVLSKLNILKNNWQSIRNELRSSDIGIASTSLDDLARFDWRDTPEKALAKLSRLFEGTKYLERTHSIFRHLSSVINYMKHWNVKRKVYISVLSSFNERFYSGGILFQCLFDTKQREVLAAGGRYDKLIEEYRPTGTSHAHPMPAYHAVGVNLGWDRLVHSMARYIKKPEKSTFVKKHSEEDTPNISWMPRRCDCLVASFDASVLRTTGIKMVADLIAHGYTAELAIDAHSIEDILRHYRDDRHSWIIVIKHGVAPDKPELKVKSIAKKEDTDMRTADLLGYLRNEFRDREEREGTARQSKAPPPPATIQKSNTSKANVDVLIAQHRSKKSNKWSIVEAAQSRSAELLSAFQSAPIAAIESKEEVMNLIKETRLSDPDSWRYFIQKMPLAERKYLQELHDLLLGYRAKWLEGKGNGDTDDVVGKAFVYNFRTGGCLLYDLES

>jgi_Coclu2_116559: spot 12

MQSTRLQDPDELEIAKEHDADKAAESETDKEGDSSESGSEDEEEHEQDIKVQFERIIAGFKKGELNIETITSNQDEYNYLDQRTNDHNPRNLLHMIVEAGKDSASALKPLVQHVIKQHCHLMEETDGAGTTPLQAAIIGGITKLVEYMCDAYDAKGNIDVVLALVDSKNNNCLHKAMLSKHKYARRIALKLIKRVRNAKTLEAQNNEFKTPLHIAVEWERCTESQMDVVRALVNACDAALNVNVGADIEPCRLSVYRYHEKTRQSAKKAEAGAKDTKEERSGNSSSDKTATSSRNTKESSKSVSGGQQVPKAPDQVRPVPTYQLNRSKEQPQGRHKDPAVEQEDLPMRGIQRQPTRLDTTAARIAHVDEFKPVGKSSVAMSPALSKAPASGTNKTSKHKEVVTEGTANAIRDFLKLYYMGSQEDHESIVSFLYGPVQEKELYWSLADGPPIISLQRLQKMSYVHFEDTLKYVLLPNIRIDKPPAAVSKFLSRSTAKDLGKGRDDYTVIFDWLRSKGVKRIFNLYIDDSEQPSHSDETIEKALQGIEVLKVWDWQRTDLSSEVIAKAAPNVSQVNLYWGGNNAVLRSWSESEGLNQLKKLDTVVLYATQGLETRAREKRNLDSFCARLKYNRSLLIESIESKQSKDSKNQEPLPPLIKVQRDAFPLKKTELGEGLTAAQEQHKDQHKWLTCMDEFATFIQNVDLDTSRLNGVEPKPIVVALIDDGLDINEQSVVDKVIGGRSFRNQNNLNAPYWATSGFHGTVMASLICRVCPMVQLYVLRLDEYSAKEQGKRQITAKSAEKAVRAAIDRGVDIISMSWTIEKTVDNEADIRKLEISLDEAAKKNILLFCSANDEATEADGSHPAANPTRFKIGAATAWGTAWRWTRASHVDFIFPGDRVIKDRPGNVPFEKCSLVSGSSVATALAAGLAALVLYCVQFSVYHRNVTNQQGKGVTLEDFQAMKSRDRMAEAFHAIYTTKDSDHKFIEVWNTFETAPREGEGKDKDTKREIIVTVANKLISRRKLVFRS

>jgi_Coclu2_21721: spot 13

MGRPKRVLSFMSQWGGGIPNNSHKNPSSAGPSPGSMSPAAPPSPSTRQPAASPNLAQSATALEAYNNDRPPSRTAGRERSDSRASVRPMSMIQTYQPPVMEVSQDTLPELQRIFMFLNSHSNKLYQEGYFLKFHDTDSRGRPAADRRWQEYFVQLVGTILSLWDASALDQAGGEGEVIPTFINLTDAAIHMIPSMTLKDGKKLDNILSVSTAASNKYLFHFDSYHALTQWTAGIRLAMFEHTTLSEAYTGALIAGKGKSLNNIRVLLDRQCAKYEDWVRVRFGAGTPWRRCWCVVSPPDEKEWIKLQKLQKKGSAYERPPVLKGDIKFYDTKKVTKKTKAIATVKDAYSCYAIYPQSKPLIDQSTLVKIEGKITVHSTPENTTEGFVFVLPESRPAISGFEIMLQFLFPVFDTFSLYGRPKKLIADVLDTRGLMFAMPSDRRYGYLEMWDVVGLINTEGSDKWTERQWRKQLKDLTSTRMMTSGSDAGSIRNRASRRNTVGRAGFTPRGLRFEDHGSIRSSPSTRQSSPVRRAHQDLEVQAPRRVGTAPAVADPSVTPRHQRSVSEQVNGRGQITPSRFVPDQASIEDENPPTPPEHRIFNGTPQNGAVQPYDSSDYDESPSREPRDLPPPPADLAAPPQGYVDLPPTQEHAAYQKPPIRPNIPAMKPNPAVDSATLQQMADAVHAPLQPGVAAWRGQETIHSRRSGDYSDQNQPMTLGVNSRPPPTNQNSGNRLSTIPASPYIEHSEFVDPPTTYQPVAPPVPEHSELPQQFESQELPFRPHPENGSIPRKPVPGPSPLASQEDDTLSTNSSRLDSLQNDVIDPEALEALDAFNVAQATLARRPSMSSSHYEDDAVSTSTPDYASIAEEESRPPPRALPQRREDRPRSGVLTFVGDPNVKGPADQDPHGYKSILDPAQAPADVPSIDFGPTYALDFNEKRPSTSGTMTQMLHDGGASRSKENLVTTPNEQKRASYISGASPPHAHSGSNSSQVNDSRQVPWQPAVPSTHQSDKQKLDPQDWVMHRASLQQPKMPGHGRSKSHTPPPFSRTHSGDWTQAQRPVHESAGVVRTPSRPLSCGAETLLDQRPTTLSAREQEQVARMTKTPLLDLSQTPKKLQKEPPMGLTGLIDNREKERAASKANRQSYSSAMQVEIDRRMLQNQQRQIMEAQQRQQQMMMQQQLQQQQLAIAQSTYAPSMMGTPSGMATPSVMGAPSMMGTPSVMGTPSVMGMPTGPGTPSNVSVMGYSTPNQMMYQQYFPQQASTPYMQVPGAWTTPTAQISQGQYFPQQGYGNMGQQQQAPTQAYGASFDQAQEAARQSYQQGHFRR

>jgi_Coclu2_22022: spot 14

MRILRRCNIFLTDAASNFRIPQLQQHYDLVAARPTDERTLQQACQSLDVDIISLDLTRRFETHFKFPMLGTAISRGIKIEICYSQGIMSHDPAAKRNLIANAVQLIRVTRGRGLIFSSEATSVLGIRAPSDVINLASVWGLGTEKGKDGLTKEARSAVEFARLKRQSFKGIVDIVHGGDKLPEKPLHETKDKQAQKGKANKNQNGKRPGESLDSSLGPETKAEKPVSKRQQAKNKKAKVQEPGQQQST

>jgi_Coclu2_116337: spot 15

MSADDKDVDPTEFVERIRRLGDQRDQEDAERVKKLEEELIQGRSERLARRAERARSISPDKPYTPQSPRSVAGTPTPKAVQDKAAANPAPPLSPPSRDAARDESLQKLTSSPPPTSTSTTMTLEDMEPKKPATSAAALGRSGTLSWKQRPQSGSIRRPLSVASRSPERPSSTNTPEPSSPEPTMSRSSIAASLGAKDPAFFRQTADGQRGSAAFRKNQDDTASETGSVSGRRQLPGMSRDSTAEQDISSPPAESARSSSPGSRGGSVRGSAVMSNRFSTSTSVSGGDSDSAVKGKTPLPVLDSQKFAPPTEQGSSVDGGDSERAARAPAMSPTQGRISPERERSVSPTKGMGGFVQSAMLKRSDSVSKRWSTQTPPGLTRQNSSLSNRGSVYGSLSNIDRPVLTRDNSMEPSRPTSSHSNATITGAEKDSAAKDEFVKPSLPRHSRAKSVASTFSATFSEKDAAHDETSPPSPSKRWSPTKSSWLESALSRPESPKPKQAPPAQPAWMAEINRIKQQRSSVDLGRASPFAEPTSSGRTSPIKDIQLRPVGLRRSESPKKEETSPTRMETLKGSPSTPAPATVPKPSSPKKEDSKPSEEIPAPEAPASSKDGPEQTSGDVPAPTVAPVTEEKQAPTDAKPVASRFAKDVGRGLPATKAESPPKTDFRAGLRSRQPISDGSKKDEVNEFQNVFGRLRKAETKNYVAPDVLKANITRGKDALNITGGPKPSVRKDEFRESLIKRKSTIFDKAQEEGSVLKKSSESSSKPSPPTPEAIAKQKGLQRRDSEARAMAPAKEKEPLPEALARRKSLGAVKAAMADNKPAQAAQAAQPAPLFAKKEPAMSGKLASRFNPALAGMLARGPPPQATDRSTSSAEVDGSSARPAQAEKTGPAPELQHMTKGRARGPKRRAPATKKAAVELEQAQEEVPAAAATALPLVNSEPVLSSSEPPKTNGKPEERRVAGTPARQSSTEKPATPAKSPRVLSGNFGKASTPELPKKPESLDLDRRVSGSQTTPKPSPKPSPRPESISSPKPSSPVIPKKPTSLESERRVSGSHSTPQKSPLPAQVETPKQSSPSPSPHSRFSRPLPTLPPKPASESPRSTPLREISTPNVEISQASDSSPEKSTFSSVKNASAMWGRSSASSSPVPTRAKSPIKLPTQADEKAAMQNAGLVRPEAESKALPDIPQPGSQSPEEVKPLPPKPKPVGLGFSLGSLGGLVASRSRESIPEPPKNAPISPPGSANRPFSEPPVASPTSKKNDGMFADFFDEAPVTEGQLPEHIDTMHILKSPPFDLGPAGKIRTLRKQMDEVTSDGKLSPIPAQEEHILFQDSMYLCTHVYDDSKGSRHTDVYLWAGNAVPEPTIEDVQLFAKNHARQSQGQLLLIRQGQETPNFFEALGGIVITRRGAKPGSKEFMLCGRRHMGHLAFDEVDFSLKSLCSAFPYLVCTSAGRVYLWKGRGCSAEEVSGARLMGMDLAITGDYNEIDEGAEPQDFLNNTFPPSPMPSKGPAIPRSADHWRYKATSDKYRTRLYKLEQTTSQQAGWGQALQVSSSFFAPLLRRPSWNAAEQRPQTPLTPKSPQGAVKTEIREIMPFSQRDLEPENIYVLDAFFEMYIIVGSLSRTQAPAFSTALMFAQAYGMLAVSEEDRPFMPVTTVVLEGVPRDMKVLFRHWDDKLIPAAGLMTGKLGRGKSLRIVGLEKAIEATRR

>jgi_Coclu2_51954: spot 16

MGVVEKPHSSPAAQRRAWTSMEEELIYYKTQYETLENELQEFQTSSKELEAELERDIEESEKRERKLQEKAERLVFEVEEWKTKYKQSKTEANNAQTKLQKEITALRESQRALQMRLRDIEVQSDDFERQARNQTSSLEDVESKYNVAIERGVMLEEEIKIGEQEREQLRIETQRLRDELTDLRIETEIVQEKLRIAEETIEGHHQRKVSSHLAGDALRPRSPMSEASTTATTLSSPTIASTPPHSIADVLHPDTTPPSPPLSDAPITAKPVPSTPMPRRKNSTAPVDPGTTPRAGMYSRAPRHSRGPSVSGSSRPPSSAASGRATPSLRPSAPGSRDPRPSLGASTSAGLPRSGSLYQIRGLIGKMQKLEQRVQTARSKLPAPTSTPPRASPRNSNVPNTVTLRSGRKRHSQVSTASSIPSAYESGVSRLSFGVSSSSKEPTSSRPGSRASLSSQPQQQHPPSRSSTQTPLGHYQSSSISGIESRVPRPRSSMSGHSNGHSHSQSFSSSMRESDASSEGSRVTTPMGRRPTMEKSGIPAPSGIPRRQSAGRRASAGQESGEMAFPVRLRRMSTQAEEESY

>jgi_Coclu2_140405: spot 17

MTSSQKRASATPSSPVSSRSFLSRFKSPLSSKSRNYTEFYIQSDDPHRQYSPGDVISGTVIIKIVKPLRITHLVVSLHGYAQVFKNPNAPGDAYKNYCSTVGAGKGNKAASYFGNGFVSLFEDEVVLCGEGRLSEGIYHFNFELEFPSKGLPSSIDFERGTISYMLNATLTRPTTISPTASCDTKVYLTDTIDIAPVPPLKPRVISLEPISRRNRNKARKPPTESAAPQTPNLANPPPAASTLEPGSAIDETEGPRSPSPSDVSYGSQRSSGNASGTEYGVRSINTATDGSPSQNGSRANAKGKTITATIDVLKSGFLRGDQIPIKVTVNHTKHIRSLKGIIITLYRLARVDMHPALPVVSNSKGDKTKSEDYYPKSRTGLGGLSLSSAGSSHTFRKDLCQSFAPLFVDPRTLTAEVKCTVRVPDEAFPTISNVPGAMISFKYYVEVVVDIQGKLTGLDRMVANAGLVNVPSTAGGSSMAGDDASGGMFSTWGGVFVDTDQIRREKGIVTALFEVIIGTKDSERNGKRKQTQQHPSYNNGTNGFPDESYTDPNGEHGYDSYYYEQDENGQYYDYGYDPAYGNAPGYEQYGAGASTSFSLPRNVPEEDQGISEKERLRRAEERLLPSRPPDAAGPSAPPGAAQHGIPASAPVLPEEDDSNPPYAAASSSSSIPAHPPPPFRVTSSPSASSAWPLAGPLQRRTTNPGEDEARTSATGSTHTVRADGRGTPVNGNENDPSKPLPPLPPQSPTEGQGSAPAYLASSSSHLPPTDDKHELQRRRLEMERSAPPMDHDEEGGPSNSDSSLSHAPPGLHLAPSAPMLNDDDEFLVEAVRPVRSRQDPPESSRRQSEALPEYQR

>jgi_Coclu2_137106: spot 18

MASHADSAHDLLDLDISTLRQFIELFPNEGLSKVITGWLSSGIAKYPLTAESDSKEAPDLSEGGVALTQDVPVSQEDCLVLMTEGLSEAPKSPLAHCLLGDYYLFLEEYESAVETTRKGLKYAAAEAKKTDLSFQKTRDALSSTLATALVYYQAPRNHLEAKGIFESILKRKPQFTSALIGIGLVLEEEEEYEQAFQFLEKALQQDPSNGRIGAESAWCQALAGDYKTGLERLQKYLDYPQLDASKQRGRELRAQTLYRIGVCLWELDPSKAARKDRQRTYAKLLAAIKANPNYAPAYTLLGIFYEDYNKDRRRARQCFQKAFELSPSEVVAAERLARLFANQGEWDIVEVVAQRVVDSGKARQTPGSKRKGISWPYSALGVVQMNKQEYQKSIVSFLSALRISPDDYYSYVGLGESYHNSGRYNSASRAFNYAENPTDGVVLKRSDEEGWFTKYMLANVNRELSEFDEALAGYEAVLAVRPKEFGVSIALLQTLVEKAWHCTETGFFGEAADSVTRGIEVADTITEYKPDAFNLWKAVGDACSIFSWVQDKLDQCPFELVVKVLQSKSDMDLDFDAHKDIDGLGQAELASLSTNRDSILTKILKAAILAQKRAIISCAHDIHAQAVAWYNLGWTEYRAHVCLEQESGKESTLTTYLRAAMKCFKRAIELEAGNSEFWNSLGVITTTLNPKVSQHAFVRSLHLNERSVHTWTNLGALYLLQNDTELAHAAFSRAQSQDPDYSLAWVGEGIIALLTGDANEALSHFTHAFELSESSLLLTKRQYAVSAFDFIASSSSSSSDITSLIQPLFALQQLIMQVPYDIPHRHLAALFLERIGNHDAAVTALDMVNEAVEQEYEKTESKKSLARVAQAKTDLARNLLAIGSDEIAVEDAETALDLLSEIESDSKQTVMSRSQLARTQLSARLTAGLAHYFYGSFDAAIAYFRKALESTGANPDIICILAEVLWAKGGENEKQVARDQLFAAIEKHDGHVGLLTLIGAMTVLDDDLETMEAIKDDLDRMRANKNLTDLQLARVEKVVEAISISLGGEEQELDEARRSVMLAPYKHTGWAELADATGGNEFASTLAKETAMRNAPPKGTLGAAGLAGAMNGTGDVGDAQRAIMLAPWRKDNWGGLAECIQSA

>jgi_Coclu2_46252: spot 19

MPPSTCLRSLSQLSLDAAPAVPRLLRPQIACFSTSSARYAVVAKKKGLTAAPKKGVKSLNTKKGKKAPGSSDTGKRPAPGERKAQRKRIVLSNDNALEVSSLQDLSKENVLSEKNEGKVMGLPQENVVDALRAVEAFKTSQGWSLFRRPAVLMRKEAIQLAKLFKEVEDSASGQKKTVRRILCGERMSGKSTLLLQGLTMGFLRDWFVINLPEAQDLVNAHTEYAPLPNSEPLQYTQDTYTANLLQQILKANGAFLQATKISTKPDLPLPLPAKATLKELVALGMANPESSWPVFMALWNELSLPGRPPVLLALDGLSHIMRHSAYLSAEVKPIHAHDLALVRHFVDHLSGQRKLANGGVVLGATSQSNSPTSPALDFSIEVAQARKVSADSVPQWNPYKSVDARVMEALKGLHDSSPDFDVIKVGGLSKEEARSIMEYYAESGMLRHRVDEGFVTEKWSLAGMGNIGELERASVRMRL

>jgi_Coclu2_64448: spot 21

MSSSSRGGSDRGRGGSDRGRGGFRGDRGGGRGGGRGDSPGRGGRGDSPGRGRGDFGNRGDFGGRGGGAQRGDRGGFRGDRDQRVLKIENTLHPPSKKTIDLSKLKLSEGNPTRPGYGTRGVRVELTANYVELLPSSSLVLYRYDVDISPDVAGRKRHRVVQLLLETAEMSAYRGSIATDFRSTIVSKTRFTHDDDIIQIQYRSEGEDEPATSAAVFKARVQYTNTLSVSELVNWMNSTDIAQSFEHKQEFTQALNIFLNHYAKSANNLATIGSSKSFSLEQNAPKADLGAGLEVIRGFFSSVRIATCRVLVNINVSHGAFYHNGPLPGLMEGYGTRNTIALEKFLRLIRVRTTHLKEKRNKANEVIPRIKTIFGLARKDDGHAMEHRPRIKQHGAGAKDVEFWLDGERSSSPKGKGKAPTQGPSASGSGRYISVYDFFRTTYNRTLQYPHLPVVNCGNRQNPVYLPAEVCVVEPGQPSKAKLDALQTQVMIRQAVRKPWENADSILKDGIRTVGLDANTNVLLQSFGLSITPGLIKVPGRILNGPQVLYKDEANAAKVSFADPRFGAWNMIKIRFNKGANLMNWKTVMIGLPGRRDAFNQPELVSLLQEFHRGLRKIGINANPPASPERLQLQHTDDPALATYLKRHSGKIELLFVILPEANIPLYKRIKTLADRDFGIHNVCAVGTKLAKERGREQYIANVALKFNLKLGGTNQEVDRKNLGIVGENKTMVVGIDVTHPAPGSASNAPSISAMVASLDYSLSQWPATLRIQRGRQEDVDDLASMLKSRLHLWKTKGKHQTFPDNILVYRDGVSEGQYDMVLSKELAQMRKACEQVYPAADTKKGLPRFTVVICGKRHKTRFYPTKKEDADRSGNTKPGTVVDRGVTEARNWDFFLQAHAALQGTAKPCHYYIVHDEIFRQNYAKQIPPPFQNVADLIEDLTHNMCYLYGRATKAVSLCPPAYYADLACDRARCYLTHLYDTPAPSAAPSVTGVSSTGDGNAPAPEPGSVEIHPNIRDTMFYI

>jgi_Coclu2_59412: spot 22

MAHLLRGKQAGISNDLSQGLSPDLFVLDHIRNYGINSKITQVAYDPVQSLIAVGTSESKYGPGQIYIFGQKRVEVVLPLPNRSASVKILQFCAEKLLCVDSKNDLSVFSLESKKLLNAHSPPAKITALHSDPAIDYALLGTQHGEVYAYDLDRETLTPFKISNLWREKFPQSRLTPVVTLSLHPRDIGSLLIGYNSGAVIYSFKQNKAIHFFNYRLPKGAPGGDSDPSSIFKDREPPLTQAVWHPTGTFILTGHEDSSIVIWDSKDGRIIQARTLTDTNVNKPGPGSFSPGAVEGSFALKSPIFKIAWCANQDPDDTAILVAGGQPSNISAKSLTLFELGRTPVYATASWQVLSDHFENPKRQRILPCPPGTEVVDLFLIPRSSPHYAGCHDPIAIIALLASGELITLSFPSGMPITPTNQLHLSMTLVHPYVNYINLAPVERTRWLGMTEKRQHGPQFLIGGAEAVHPLKRFENRNIVQTAHADGTIRLWDAGHADEIENHALLQVDVARAVGRVEDVHVTQTSFAGAASELSVGLKTGEVVVFRWNVNSRAGQDHPPGENTPQALTNITDRCEPTVKEGLLPFTLFDGSNGSVTALKHSDVGFVAAGFEGGSFAIIDLRGPAVIFQASTKDFIKSDKRSSFRRNSTQAVPKAEWPTCIEFSVMTLEDEDYSSILVHVGTNLGHLATFKLLPESSGRYSAKLAGVCSLDDRVQSICPIHAETGRPAYASQSTVGGLRTGLKVNGVLLAVTCSETRLFRPATSKGAHKTFDQFLCDTAMVVRYEQSGYALLGLYGDGCARAYSLPALKEIASVNVSDFLDVRRFSEAIITATGDILGWKGPAELSLVNIFGTGFRYDRTKDVLLNPDLVIPPRPTISNMQWISGTQYVTPADMDLLIGGPDRPLSNRQLAQQRAEAEQERRRNHPSSSLSPAAQYQSYPTQANSQTQEGWGAWASRQLNERTEKLNIVGDSMDNLSQNSAGWANDVNKFVGKQKRGLVMGAVKSKFGL

>jgi_Coclu2_29439: spot 23

MANIEQTWSLAGKVAVVTGSGRGIGKAMAIELAKRGAKVAVNYANAVEGAEQVVKEIKALNNGSDAHAFKANVGNVEESEKLMDDVVKHFGKLDICCSNSGVVSFGHFKDVTPEEFDRVFNINTRGQFFVAKAAYKRMENYGRIILMGSITGQAKGVPKHAVYSGSKGAIETFTRCMAIDAGEKKITVNCVAPGGIKTDMYHAVCREYIPGGEKLSDDQVDEYACTWSPHNRVGQPVDIARVVCFLASQDGEWVNGKVIGIDGAACM

>jgi_Coclu2_58690: spot 24

MADERSPLIQRVDVRPHRDRYPHHRLRFFCTTLLSATIVAGGLAAVFFLSIPPPNENEEASLSRVPTFLSSSTSQIPEGWARKTTLGFDDLQKTLLETPNAETAEKWSRYYTSGPHLAGKNLSQALWTRERWQEWGVPSTIVDYEVYINYPKGHRLALIEKAEPGHEKKDTSAASENTWKVTYEARLEEDVLDEDKSSQLADRVPTFHGYSASGNVTAPYVFVNYGTYQDFEELRTANVSLEGKIALAKYGGVFRGLKVKRAQELGMVGVVMYSDPGDDGEVTEKNGIPTYPNGPAREPSSVQRGSVQFLSFAPGDPTTPGYPSKPDCPRQPVDHAMPHIPSLPISYLDALPLLKALNNHGPKASSFSNHWHGGGLDYKGVQYNIGPSPDDLVLNLVNEQEYVTTPLWNVIGVINGTISDEVVILGNHRDAWIAGGAGDPNSGSAALNEVIRSFGAAVEAGWKPLRTIVFASWDGEEYGLVGSTEWVEEYLPWLSKSAVAYLNVDVGSVGPDFKLAAAPLLSRVVESAIQMVASPNQTVPGQSVYSVWDKLIETMGSGSDFTAFQDFAGIPSIDMGFGYNPKSAVYHYHSNYDSFDWMKKYGDPSFEYHAAIAKAWALVAARLIDLPILQLNATDYALGLRKYVQAAKHTAHASSPDVSPEIWQPLDEAVARFIKASTVHDEYAAYLQDVYSRIDSIPWWQPWKKVQLFLAIRAVNTKYKYLERKFLFPDGLDGRSWFKHVVFAPGKWTGYSGATFPGIVEAIGEKDEDATERWIDIVSKAIGSAADWLEKDEQAKLNF

>jgi_Coclu2_125654: spot 25

MAAETYSGVAAIIRNLARQHDPERDPSFSAQVSANGAKTAAIALPGPDSEEKTQLEQELSALCSRIDFLEQKCSQAAAKPGQLPLTPAQEAPDDGVLCTPAGAIRNSSAPPGQARRGSNKERAIWVSNWLAAKESNGTTEEPAAALTEEQLNYLRVHLNQQADQIRNQREHIDNLSREVNKQLTTQSMVFEHGIEDIGALKRELGKHQQANLAFQKALREIGAIVTAVAMGDLSKKVLIHAKEMDPEITLFKRTINTMVDQLQEFASQVTFLAREVGTEGRLGGQANLPGVAGIWAELTDSVNMMANNLTEQVREIAVVTTAVAHGDLSRKIERPARGEILQLQQTINTMVDQLQSFATEVTKVARDVGTEGKLGGQAEIAGVKGMWNELTVNVNAMAQNLTTQVRDIAQVTTAVAQGNLTRKVEAQCKGEILELKNTINRMVDQLQQFAHEVTKIAREVGSEGRLGGQATVHGVEGTWKDLTENVNGMAMNLTTQVREIAEVTTAVARGDLSRKVKAEVQGEILSLKITINTMVDRLNTFAQEVSKVAREVGTDGILGGQAQVDNVEGKWKDLTNNVNTMAQNLTLQVRSISEVTQAIAKGDMSRRVHVDAEGEIRLLKDTVNDMVMRLDEWSLAVKRVARDVGVDGKMGGQADVRDIDGRWKEITTDVNTMAQNLTSQVRAFGDITNAAMEGKFTQITVEASGEMDELKRKINQMVSSLRESIQRNTAAREAAELANKTKSEFLANMSHEIRTPMNGIIGMTQLTLDTDLTHAQREMLTIVHNLAGQLLTIIDDILDISKIEANRMVMEEIPFSMRGTIFNALKSLASRANERKLNLAYDVSYTVPDYVVGDSFRLRQIILNLVGNAIKFTEHGEVKVAISMASEQDCGPDHYVFQFAVSDTGIGIRGDKLNLIFDTFQQADGSTTRKFGGTGLGLSISKRLVTLMGGRMWVESDFGKGSVFYFTCRVRLGKPEITAIQPQLVAYKGHTVLFVDQGNTGFSDQIIEHLKALDLVPMVVNSVEEVPETTKRADMPYDCVIVDNDKTARELRIAERFKYIPLVMLTPHVCISLRSALENGISSYMTTPCLPIDLGNALIPALDGRAAPLVSDHSKSFQILLAEDNAVNQKLAVRILEKYHHRVTVANNGLEAFEHIQKKRYDCVLMDVQMPVMGGFEATAKIREWERENGIPSTPVIALTAHAMVGDREKCLAAQMDDYLSKPLRQNQLIQTILRCATVGSTIYDHTSEPRYTAPSHIPNLELPSNSKDQANTGAPSSSSTTSPKKRPQLEARGFTDRGAGADSPNLLAVDQTDNGSVERVRSLSASL

>jgi_Coclu2_132047: spot 27

MGFSLNNPLPSSMSSECRKTGKILASFVDPRQAFGPDKIIPPSVLANAKGLAILTVFKAGFLGTARFGSGVVVARLADGSWSAPTAIGTIGGGFGGQIGFELTDFVFILNDAAAVRTFAQAGSLTLGGNVSIAAGPVGRNAEAAGAASLKSVAGIFSYSKTKGLFAGVSLEGSGIIERRDANEKLYGRRWTAREILSGQVPPPPAAEPLLRVLNSRVFSGVGGAHNVTNDAMYNDIPVYDDAHDNVVWQGRRGSAMGEGVRRDRTGSMGQQDDYVYRDRPQHSSTWADDVYDRQPSSAPGGLNRSFSTRANPTETFDRIDSRNRASTFGDDYSYSDRKPGRPSAPKPVFGQTTGQKKAGLQSNQAIAKFTFDADQPGDLGFKKGEIITIVKRTDNDTDWWTGRIGDREGIFPSNYVETV

>jgi_Coclu2_123093: spot 28

MVQASEVLSRKTGVIVGDDIHKLFKHAQSEGYAIPAINVTSSSTVVAALEAARDAKSPIILQLSNGGAAYFAGKGVSNSNQEASIAGSIAAAHYIRSIAPAYGIPVVLHTDHCAKKLLPWLDGMMDADEAYFKQHGEALFSSHMIDLSEEPKEWNIATTKKYLQRAAPIKQMIEMEIGITGGEEDGVNNEDVDNNSLYTQPEDIYDIYKELSEVSPLFSIAAGFGNVHGVYKPGNVKLRPELLQKHQAYVKEKTGAKDDKPVFLVFHGGSGSSVDDFRQAISYGVVKVNLDTDMQWAYLSGIRDYIQSKSGYLQTQVGNPDGDDKPNKKYYDPRVWVREGEKTMSQRIKVALDDFYTAGKA

>jgi_Coclu2_16455: spot 29

MTLSSPLPSFSSPASSRLPAARRASFSGQLAFLVHSQETVANHMPPDVDNKALARQKRRRTSKEDEDILKSEYLKNPKPSKAARLEIVKKVALGEKEVQIWFQNKRQNDRRRSRPLEPSSTASFLSSSSAMSDPLTEDEVMPGARRIDNAPEPKESDECLKSDPPEQPATPELTSDETIPEPRTIDTALSVAAESERANETAVQPSTEAQAETAPVQTTDSQQAVSSSQGATQSRTSWISNRRSASFVRYLEDYTPEVITFPNAPPRPSESPETTAKTPSRPLKRTHSFMRLSTNENGTARIVTDLDKTPSPPKSKKTPSSFSRTAAGLRRSYSAAGLNDRLAAAARGEPTPKIPRTVSSIGRSRDSRAWEFWCDPETRSTTSLTARAEQEESGSAADAIGILRANRRILARNQARQNSSPLTGRHSLHKVLGTPSVKKSRGPMQRASTISGRLSHNDYSDYKKGGDSTESDEFPQTESDKENWEPNAPKTVCRDRQVAATPPASRGARQILGENRELMSQSSSLGALLAKEKRSGGKRVIDPEQDDELRQFMNGDGASGRSSINSAEEAGCVEGLLKLSQGQWR

>jgi_Coclu2_114571: spot 30

MANITISNQPSEYLRVFDSRTCNIHHIPIVDGFVRGSDLSTIIGPVNGSKGRLQKLAVLDPGFQNTACKESDITFIDGENGILLYRGVRIEDLFHNYDFDATLHLLVWGRLPNSEEKNGFEQRLAKAAHPPQEVCDVIRTLPRNMDFVSMFLTGLSTYMGTDDEMTKSRHQAVMTYHNNLPRTDDAIIRCFAYVSTTVAICYCHITNVEFHTPQQGLTLVENFLHMTGMEDPDKRISRTIDRLSMNLADHGLSCSTAAFLHVASSMTDPMTCLLAAISAGSGPLHAGALEICYQELERIGSVDNVPAYIAGVKTKKFRLFGYGHRVYKTQDPRAALTKELLEEHREAIEANPLLQIAIEIDKQANTDPYFVERKLKLNADFYGCFIYIALGIPREVIPGLLTISRMGGFMAHWRESMTNPMKLWRPMQKYKLRTS

>jgi_Coclu2_141118: spot 31

MSAEPDHTKPKPDLSKNPDGAEPKENDETATAILKKKKKPNSLIVTDAVNDDNSIIALSNNTMETLQLFRGDTVLVKGKKRKDTVLIVLADDDLDDGSARINRVVRHNLRVKHGDVITIHPCPDIKYAKRIAVLPIADTVEGITGSLFDVFLAPYFREAYRPVRQGDTFTARGGMRQVEFKVVEVDPPEFGIVAQDTVIHCEGEPIQREDEEGNLNEVGYDDIGGCRKQMAQIRELVELPLRHPQLFKSIGIKPPRGILMYGPPGTGKTLMARAVANETGAFFFLINGPEIMSKMAGESESNLRKAFEEAEKNSPAIIFIDEIDSIAPKREKTNGEVERRVVSQLLTLMDGMKARSNVVVMAATNRPNSIDPALRRFGRFDREVDIGIPDPTGRLEIMQIHTKNMKLADDVDLQTIAAETHGYVGSDLASLCSEAAMQQIREKMDLIDLDEDTIDAEVLDSLGVTMENFRFALGVSNPSALREVAVVEVPNVRWEDIGGLEDVKRELIESVQYPVDHPDKFLKFGMSPSRGVLFYGPPGTGKTLLAKAVANECAANFISIKGPELLSMWFGESESNIRDIFDKARAAAPCVVFLDELDSIAKSRGGSQGDAGGASDRVVNQLLTEMDGMTSKKNVFVIGATNRPEQLDNALCRPGRLDTLVYVPLPDLASRVSIIKAQLRKTPVADDVDIEFIAQNTHGFSGADLGFVTQRAVKLAIKQSIAIDIERRKAREAAGEDVDMEVDEEDPVPVLTKAHFEEAMRSARRSVTDVEIRRYEAFAQSMKNSGGSSFFRFPDAENAAAGAEQNTFGAGGEDEDLYN

>jgi_Coclu2_44339: spot 32

MSAPIEGQPESPPTASQLSSHPALHPDITSIPVTPGVHLSGKTAYFDESADKKQDNSLPFDPETATPGPSWSANSYFSNQQRADDAGPRADKAAGAQTGAEVKNKLNETSGSASKANLADVDPRAAHPGLNLSGRVISATFAIPYTIGYSPDSDWELSPRRGTSALFDSFSYLASSSSPWNHTLVGWTGEISHAATAAQPPTNKAAAPIPVDPKDPKQATQASNGFRIGPEDRARLEKQLERDHGGRIVPVWLVDEIDDGKDEYILKNQSHWRTYAEHELYTLFHYKQNEPADGRAARKSWADYYRMNRLFADRILEIYKPGDIVMVHDFYLMLLPSLLRQRVPNIYIGFYLHIPFPSSEFYRCLSRRKEVLEGVLGANMIGFQSYSYARHFSSCCTRILGFDSSSEGVDAYGAHVAVDVFPIGINAVSTQRQAFDDPEIEEKLKGIRELYAGKKLIVGRDRLDAVRGVVQKLQAFQLFLEKYPEWQGKVVLIQVTSPSGLHTDKSDGAQEKVVNKISDLAAKINGTYGTLDFTPVRHFPQYLSREEYFALLRIADIGLITSVRDGMNTTSMEYIICQKDNHGPLILSEFSGTSSSLGSATHINPWDMSGVADAINEALKQDEAERTRVHAELYKHVVENNVQAWTNNYLKKLMRNLSSFDQTFATPALDRAKLLFQYRQAKKRLFMFDYDGTLTPIVKDPQAAIPSDRVIRTLKTLAADPTNAVWIISGRDQAFLDEWMGHIPELGLSAEHGSFMRPPRSQDWENLTETTDMSWQNEVLDIFQHYTERTQGSFVERKKIALTWHYRRADPEYGAFQARECQKHLERTVAKKYEVEVMTGKANLEVRPRFVNKGEIAKRLVEEYGDGPGEAPEFVLCMGDDFTDEDMFRSLRQSKLPTDHVFSVTVGASSKQTLASWHLVEPSDVISVISLLNGSADAGNVGAVAIVDGSVPESRAGP

>jgi_Coclu2_46026: spot 38

MKNRTVMNYVFSVIVGFVAITYGSVPMYKMICQQTGWGGQPIKSAAHGGDSSVDPAERLKPVVDHPRIRITFNGSVSDVLPWKFVPQQREVRVLPGETALAFYTATNKSPEDIIGVATYSVTPGQVAPYFSKIQCFCFEEQRLNAGETVDMPVFFYIDPEFVTDPNMKGIETVTLSYTFFKAKYDKDGHLRPVPMA
