# Supplementary material for: Secretome weaponries of Cochliobolus lunatus interacting with potato leaf at different temperature regimes reveal a CL[xxxx]LHM - motif
Source: BMC Genomics. 2014 Mar 20;15:213. doi: 10.1186/1471-2164-15-213 (PMC4000054; doi:10.1186/1471-2164-15-213)
Supplement: Additional file 2: Table S1 — Details of primers sets used in semi-quantitative RT-PCR. [file 1471-2164-15-213-S2.doc]

| **Spots#** | **Sequence accession** | **Primer Sequence** | **Tm (°C)** | **Size (bp)** | ***Cycles** | **Putative name** |
| --- | --- | --- | --- | --- | --- | --- |
| 3** | jgi_Coclu2_118437 | F:ACACCTCCGTCCTCCA | 55.7 | 196 |  | unknown (ClEfc1) |
|  |  | R:GCCTGGGAGTTGAAGGA |  |  | 30 |  |
| 6** | jgi_Coclu2_19257 | F:TTCCTCTTCGCCAAGGA | 55.2 | 215 |  | unknown (ClEfc2) |
|  |  | R:AGGTGGTCGGACTGGA |  |  | 30 |  |
| 19** | jgi_Coclu2_46252 | F:CCAGTACACCCAGGACA | 55.3 | 211 |  | unknown (ClEfc3) |
|  |  | R:GGAGAGCTCGTTCCAGA |  |  | 30 |  |
| 21** | jgi_Coclu2_64448 | F:CCCCTCCAAGAAGACCA | 55.1 | 213 |  | unknown (ClEfc4) |
|  |  | R:AGGAGGAGCTGGACGA |  |  | 30 |  |
| 27** | jgi_Coclu2_132047 | F:TCGCCTCCTTCGTCGA | 56.5 | 252 |  | Unknown (ClEfc5) |
|  |  | R:TGAAGACGAAGTCGGTGA |  |  | 30 |  |
| 7 | jgi_Coclu2_115068 | F:ACCACCCAGTGGTCCA | 55.5 | 167 |  | Heat shock protein 70 (HSP70) |
|  |  | R:AGCTTGAGCTCCTCGAA |  |  | 25 |  |
|  | Contig_10228:29-375 | F:CCGGTCCTTCTTGGACA | 55.7 | 167 |  | Scytalone dehydratase (SDR) |
|  |  | R:GCTGGTGGTATCCCGTA |  |  | 28 |  |
|  | Contig_10228:29-315 | F:CCGGTCCTTCTTGGACA | 55.7 | 167 |  | 1,3,8-Naphthalenetriol reductase (NTR) |
|  |  | R:GCTGGTGGTATCCCGTA |  |  | 25 |  |
|  | Contig_49:1-588 | F:AACACTGCACCGACCA | 55.4 | 167 |  | Glyceraldehyde 3-phosphate dehydrogenase (GADPH) |
|  |  | R:ACGTCGTTGTGCTCGA |  |  | 30 |  |
| 18 | jgi_Coclu2_137106:1-353 | F:CTCGAGGAGGAGGAGGA | 55.7 | 167 |  | Tetratricopeptide (TRP) |
|  |  | R:TGGGGGTAGTCGAGGTA |  |  | 30 |  |
| 4 | jgi_Coclu2_125544 | F:AGGAGGAGCCCATGGA | 55 | 170 |  | Oxidoreductase (OXR) |
|  |  | R:GAGGAGTCGGACTTGGA |  |  | 25 |  |
| 12 | jgi_Coclu2_116559 | F:TTCAAGACCCCCCTCCA | 56 | 167 |  | Subtilisin protease (Pte) |
|  |  | R:CGGGTCTTCTCGTGGTA |  |  | 30 |  |
| 17 | jgi_Coclu2_140405 | F:GAGGTCGTCGTCGACA | 55.4 | 167 |  | Arrestin-like protein (ALP) |
|  |  | R:GGTCGGTGTCGACGAA |  |  | 28 |  |
| 23 | jgi_Coclu2_29439 | F:ACGCCTTCAAGGCCAA | 55.4 | 162 |  | Short chain dehydrogenase (SCD) |
|  |  | R:TGAAGACGCGGTCGAA |  |  | 30 |  |
| 38 | jgi_Coclu2_46026 | F:TTCTACACCGCCACCAA  R:TCCTCGAAGCAGAAGCA | 55.6 | 165 | 30 | Cytochrome C oxidase (CCO) |
| 22 | jgi_Coclu2_59412 | F: CGTCGACCTCTTCCTCA  R: GACGAGGGTCATGGAGA | 55.3 | 166 | 30 | WD40 repeat |
| 15 | **jgi_Coclu2_116337** | F: GCCGTCAAGACCGAGA | 55.6 | 153 |  | Gasolin-like related protein (GLrP) |
|  |  | R: ATGAGGGCGGTGGAGA |  |  | 28 |  |

**Additional file 2: Table S1: Primers sequence list for semi-quantitative RT PCR**

**Entries tagged with Asterisk are secreted peptides having no catalytic domain and are potential candidate effectors.

*Cycles refer to the PCR amplification cycles above which saturation occurred.
