# Supplementary figures and images for: Secretome weaponries of Cochliobolus lunatus interacting with potato leaf at different temperature regimes reveal a CL[xxxx]LHM - motif
Source: BMC Genomics. 2014 Mar 20;15:213. doi: 10.1186/1471-2164-15-213 (PMC4000054; doi:10.1186/1471-2164-15-213)

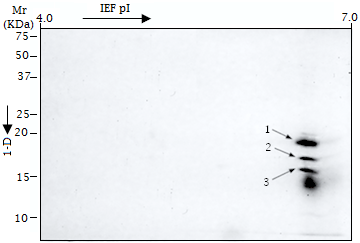

Supplement: Additional file 3: Figure S1 — A 2-D secretome map of C. lunatus in CDB cultured at 20°C without potato leaf. Identified spots are indicated with arrows. The immobilized pH gradient scale and standard molecular weight scale are shown. [file 1471-2164-15-213-S3.tiff]

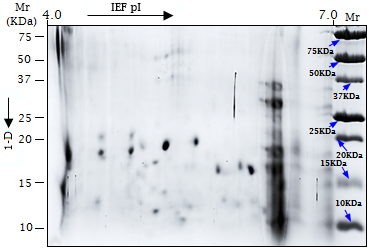

Supplement: Additional file 4: Figure S2 — A 2-D secretome map of C. lunatus interacting with potato leaf in CDB medium cultured at 28°C. The immobilized pH gradient scale and standard molecular weight scale are shown. [file 1471-2164-15-213-S4.tiff]

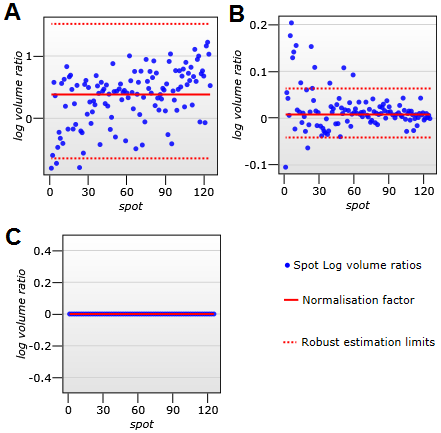

Supplement: Additional file 5: Figure S3 — Dispersion of positional constraint normalized spot volume plots generated in Progenesis SameSpot 4.1 suite. The graphs depict the distribution of spots against a reference gel for C. lunatus secretome (in this case, Figure 4). (A) Secretome plot at 20°C with leaf and normalization factor is 2.1. (B) Secretome plot at 38°C with leaf and normalization factor is 1.38. (C) Reference gel plot obtained here is considered as 20°C with leaf use for normalizing the alignment and the normalization factor is zero. [file 1471-2164-15-213-S5.tiff]

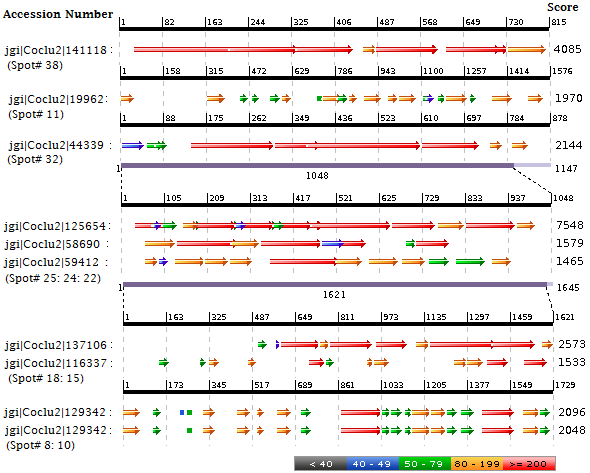

Supplement: Additional file 7: Figure S4 — Ten best peptide spots hit maps corresponding to unique protein models predicted from C. lunatus m118 v.2 genome peptides available at http://genome.jgi.doe.gov with E-values ≤10-5. Open reading frame map are indicated with broken arrows in the forward (left to right) direction with each hit associated with the protein accession number and protein score. [file 1471-2164-15-213-S7.tiff]

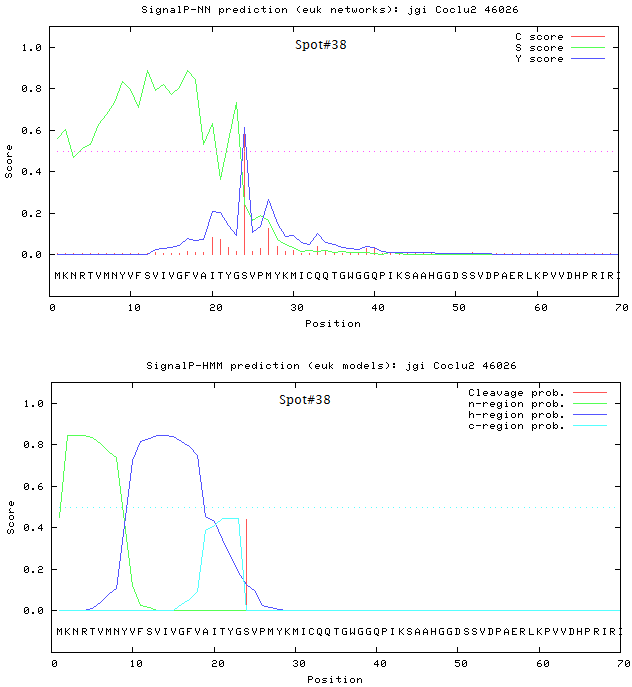

Supplement: Additional file 8: Figure S5 — Signal peptide and transmembrane network prediction profile for spot# 38 in SignalP4.1 [22]. Spot# 38 (jgi_Coclus-46026) was identified as cytochrome C oxidase. Discriminatory cut-off value D = 0.5 indicates the presence of signal peptide with transmembrane network. [file 1471-2164-15-213-S8.tiff]
