# Supplementary material for: Secretome weaponries of Cochliobolus lunatus interacting with potato leaf at different temperature regimes reveal a CL[xxxx]LHM - motif
Source: BMC Genomics. 2014 Mar 20;15:213. doi: 10.1186/1471-2164-15-213 (PMC4000054; doi:10.1186/1471-2164-15-213)
Supplement: Additional file 9: Table S3 — Details of motifs hit scores generated in MAST [29] and FIMO [30]. The likelihood scores of motifs in 23 fungal genome peptides and data on the strength of motif CLxxxxLHM tested against 23 fungal genomes at P < 1e-4 are depicted. [file 1471-2164-15-213-S9.doc]

**Additional file 9: Table S3: Details of motifs hit scores generated in MAST [29] and FIMO [30]**

|  |  | **Motifs likelihood hit scores in fungal genome peptides reported in MAST [29]** | | | **Motif 02 occurrences in fungal genome peptides reported in FIMO [30]** |
| --- | --- | --- | --- | --- | --- |
| **Sl** | **Organism** | **Motif 01** | **Motif 02** | **Motif 03** | **Motif 02**  **CLxxLHM** |
| **1** | *Candida dubliniensis CD36 uid38659* | 0 | 0.33 | 0.22 | 281 |
| **2** | *Candida glabrata* | 0 | 0.33 | 0.22 | 264 |
| **3** | *Cryptococcus gattii WM276* | 0 | 0.33 | 0.22 | 354 |
| **4** | *Cryptococcus neoformans var JEC21 uid10698* | 0 | 0.33 | 0.22 | 361 |
| **5** | *Debaryomyces hansenii CBS767 uid12410* | 0 | 0.33 | 0.22 | 324 |
| **6** | *Encephalitozoon cuniculi uid155* | 0 | 0.33 | 0.22 | 68 |
| **7** | *Encephalitozoon intestinalis ATCC 50506 uid51607* | 0 | 0.33 | 0.22 | 56 |
| **8** | *Eremothecium cymbalariae DBVPG 7215* | 0 | 0.33 | 0.22 | 190 |
| **9** | *Eremothecium gossypii uid10623* | 0 | 0.33 | 0.22 | 203 |
| **10** | *Kazachstania africana CBS 2517* | 0 | 0.33 | 0.22 | 252 |
| **11** | *Kluyveromyces lactis NRRL Y-1140 uid12377* | 0 | 0.33 | 0.22 | 260 |
| **12** | *Lachancea thermotolerans CBS 6340 uid39575* | 0 | 0.33 | 0.22 | 262 |
| **13** | *Myceliophthora thermophila ATCC 42464* | 0 | 0.33 | 0.22 | 418 |
| **14** | *Naumovozyma castellii CBS 4309* | 0 | 0.33 | 0.22 | 254 |
| **15** | *Naumovozyma dairenensis CBS 421* | 0 | 0.33 | 0.22 | 260 |
| **16** | *Pichia pastoris GS115 uid39439* | 0 | 0.33 | 0.22 | 256 |
| **17** | *Saccharomyces cerevisiae uid128* | 0 | 0.33 | 0.22 | 204 |
| **18** | *Schizosaccharomyces pombe uid127* | 0 | 0.33 | 0.22 | 204 |
| **19** | *Tetrapisispora phaffii CBS 4417* | 0 | 0.33 | 0.22 | 259 |
| **20** | *Thielavia terrestris NRRL 8126* | 0 | 0.33 | 0.22 | 470 |
| **21** | *Torulaspora delbrueckii CBS 1146* | 0 | 0.33 | 0.22 | 230 |
| **22** | *Yarrowia lipolytica CLIB122 uid12414* | 0 | 0.33 | 0.22 | 319 |
| **23** | *Zygosaccharomyces rouxii CBS 732 uid39573* | 0 | 0.33 | 0.22 | 260 |

Motifs best hits in fungal genome peptides as reported in MAST [29] at P<e^-4^ and Best matching occurrences of motif 02 reported in FIMO [30] at *P*<1e-4.
